# Indoleamine 2,3-dioxygenase 1 promotes osteosarcoma progression by regulating tumor-derived exosomal miRNA hsa-miR-23a-3p

**DOI:** 10.3389/fphar.2023.1194094

**Published:** 2023-05-22

**Authors:** Dan Yang, Yinxian Chen, Zhen Ning Tony He, Yichen Wang, Chenghui Ke, Yi Luo, Sun Wang, Qichao Ma, Mengjie Chen, Qing Yang, Ziming Zhang

**Affiliations:** ^1^ Department of Orthopedics, Shanghai Children’s Hospital, School of Medicine, Shanghai Jiao Tong University, Shanghai, China; ^2^ NHC Key Laboratory of Medical Embryogenesis and Developmental Molecular Biology & Shanghai Key Laboratory of Embryo and Reproduction Engineering, Shanghai, China; ^3^ State Key Laboratory of Genetic Engineering, School of Life Sciences, Fudan University, Shanghai, China

**Keywords:** osteosarcoma, indoleamine 2,3-dioxygenase 1, exosome miRNAs, tumor immunity, immunotherapeutic target

## Abstract

**Background:** Osteosarcoma (OS) is the most common primary malignant tumor originating in bone. Immunosuppressive enzyme indoleamine 2,3-dioxygenase 1 (IDO1) participates in tumor immune tolerance and promotes tumor progression, while the study of IDO1 in OS is limited.

**Methods:** Immunohistochemistry analysis was performed to test the expression of IDO1 and Ki67. The relationship between IDO1 or Ki67 positive count and clinical stage of the patient was analyzed. Laboratory test indexes including serum alkaline phosphatase (ALP), lactate dehydrogenase (LDH), white blood cell (WBC) count and C-reactive protein (CRP) at diagnosis of OS patients were collected. The relationship between positive count of IDO1 and Ki67 or laboratory test indexes was analyzed by Pearson’s correlation analysis. IDO1 stably overexpressed cell lines of these cells (MG63 OE, 143B OE and hFOB1.19 OE) were constructed and validated by Western blot and Elisa. Exosomes were isolated from conditioned culture media of these cells and were identified by Zetaview nanoparticle tracking analyzer. Next-generation sequencing was conducted to identify miRNAs enriched in exosomes. Differentially expressed miRNAs (DE miRNAs) were verified in clinical samples and cell lines by qPCR. Biological processes and cell components analysis of DE miRNAs was conducted by GO enrichment analysis using the protein interaction network database.

**Results:** Immunosuppressive enzyme IDO1 was highly expressed in tumor tissues. 66.7% (6/9) of the tissues showed moderately or strongly positive immunostaining signal of IDO1, and 33.3% (3/9) were weakly positive. The expression of IDO1 was positively related to Ki67 and associated with prognostic-related clinical features of OS patients. Overexpression of IDO1 significantly affected the exosome-derived miRNA subsets from MG63, 143B and hFOB1.19 cells. A total of 1244 DE miRNAs were identified, and hsa-miR-23a-3p was further screened as key DE miRNA involved in the progression of OS. GO analysis of target genes of the DE miRNA results showed that target enrichment in the functions of immune regulation and tumor progression.

**Discussion:** Our results indicate that IDO1 has the potential to promote the progression of OS that is related to miRNAs mediated tumor immunity. Targeting IDO1-mediated hsa-miR-23a-3p may be a potential therapeutic strategy for OS treatment.

## 1 Introduction

Osteosarcoma (OS) is the most common bone malignant tumor in children and adolescents (Morrow and Khanna, 2015; Taran et al., 2017). Although the 5-year survival rate of OS patients had increased from 20% to 30% in 1990s to 50%–70%, thanks to the combined treatment of immunotherapy, neoadjuvant chemotherapy, and surgery, there was no significant breakthrough in the past 10 years (Longhi et al., 2006; Kager and Bielack, 2017). In particular, the curative effect of patients with relapse, metastasis, incomplete surgical resection, or chemotherapy resistant OS has not been improved in recent 30 years, and the survival rate of patients bearing these tumors is less than 20% (Longhi et al., 2006; Koirala et al., 2016). Therefore, it is urgent to determine the molecular mechanism of OS survival, immune evasion and metastasis in order to promote the development of new therapeutic strategies.

Indoleamine 2,3-dioxygenase 1 (IDO1) has been proposed to be the target molecule of OS immunotherapy (Urakawa et al., 2009; McEachron et al., 2018; Zheng and Wan, 2018). It has been found that immunosuppressive IDO1 and fork head box P3 (Foxp3) are expressed in OS tissues, and IDO1 expression is closely related to the poor prognosis of OS patients (Urakawa et al., 2009; Zheng and Wan, 2018). IDO1 is the first-rate limiting enzyme outside the liver that catalyzes tryptophan (Trp) catabolism via the kynurenine pathway (KP) (Takikawa et al., 1986). Overexpression and activation of IDO1 by tumor cells and immune cells reduces CD8^+^ effector T cells and accumulates Foxp3+ regulatory T cells in the tumor microenvironment by depleting essential amino acid Trp and generating toxic metabolites such as kynurenine (Kyn), which ultimately leads to tumor immune escape (Takikawa et al., 1986; Routy et al., 2016). The ratio of CD8^+^ cells to Foxp3+ cells has been proposed to be a prognostic marker as patients’ groups with long survival showed distinguish data to those who are not so fortunate, and has been predicted to be related to the expression of IDO1 (Fritzsching et al., 2015), but the specific molecular mechanism is unknown.

MicroRNAs (miRNAs) are evolutionary conserved, small non-coding RNA molecules of 21–23 nucleotides (Jamali et al., 2020; Ebrahimi et al., 2021). In the extracellular space, miRNAs either bind to proteins (Vickers et al., 2011) or serve as a major RNA component of exosomes (Redis et al., 2012). Exosomes are small 30–150 nm membrane vesicles that are delivered into the extracellular environment by different cell types, including cancer cells (Zhou et al., 2014). Exosomes reflect the expression patterns of dysregulated miRNAs in cancer cells. Dysregulation of miRNAs has been associated with multiple malignant tumors, such as OS (Adams et al., 2014; Bottai et al., 2014). It has been already reported that many miRNAs are expressed differently in OS tissues and cell lines compared to normal cells, and these miRNAs are deeply implicated in multiple steps of tumor occurrence and development, including proliferation, adhesion, invasion and metastasis (Nugent, 2014). Thus, we hypothesized that tumor-derived exosomes may deliver miRNAs to host recipient cells to modify gene expression on a genome-wide scale. Moreover, miRNA-targeted treatment approach has shown enormous potential in controlling aggressive biological behavior of OS (Miao et al., 2013). However, there are no reports that immune checkpoint IDO1 may shape the cancer immune landscape in the TME and promote tumor progression by interaction with the OS-derived exosome miRNAs.

The purpose of this study is to screen for the abnormal changes in miRNA expression in exocrine cells of different types of OS and normal osteoblasts by sequencing technology, to analyze and explore the maintenance of malignancy in IDO1-high OS, and to provide new evidence for the diagnosis and treatment of OS.

## 2 Materials and methods

### 2.1 Cell culture

OS cells (MG63 and 143B) and hFOB1.19 cells were bought from the cell bank of the Chinese academy of sciences (Shanghai, China). Cells were authenticated by short tandem repeat analysis and passaged for fewer than 6 months before experiments. All cell lines were tested to be negative for *mycoplasma* contamination and were cultured in an atmosphere of 5% CO_2_ and 90% relative humidity. MG63 and 143B cells were cultured in DMEM (Gibco, United States of America) supplement with 10% (vol/vol) fetal bovine serum (FBS, Gibco, United States of America), 100 μg/mL penicillin and streptomycin (Gibco, United States of America) at 37°C. The hFOB1.19 cells were cultured in DMEM/F12 (Gibco, United States of America) supplement with 10% (vol/vol) FBS (Gibco, United States of America), 1% (vol/vol) nonessential amino acid solution (Gibco, United States of America), 0.3 mg/mL G418 and 100 μg/mL penicillin and streptomycin (Gibco, United States of America) at 33.5°C.

### 2.2 Clinical specimens

Tumor specimens of OS patients and control non-tumor specimens of polydactyly patients were obtained from Shanghai Children’s Hospital Affiliated to Shanghai Jiao Tong University School of Medicine. All samples were collected with the donor being informed completely and with their consent. The procedures were approved by the Institutional Ethical Review Board of the Shanghai Children’s Hospital (2021R095-E01, 2020R159-E02 and 2020R029-E03).

### 2.3 Stable transfection with the lentiviral vector

Lenti-GFP containing an IDO1 overexpression sequence and its negative control sequence (NC) were purchased from Shanghai HuaGene Biotech Co., Ltd. Cells were transfected with lenti-GFP-IDO1 or lenti-GFP-NC. Polyclonal cells with green fluorescent protein signals were purified for further experiments using a fluorescence-activated cell sorting flow cytometer.

### 2.4 Western blotting

Western blots were performed as described (Yang et al., 2019; Yang et al., 2022) using the following antibodies against IDO1 (ab211017, Abcam), Flag (ab205606, Abcam), CD63 (ab217345, Abcam), CD81 (ab109201, Abcam), RUNX2 (ab23981, Abcam), Calnexin (#2433, CST) and GAPDH (AF2823, Beyotime). The whole protein was extracted by RIPA Lysis Buffer (Beyotime Biotechnology, China), and the concentration was detected by a BCA protein assay kit (Beyotime Biotechnology, China). Cell lysates were kept on ice for 30 min and centrifuged at 16,000 g for 3–5 min at 4°C. Supernatants were collected and boiled in SDS loading buffer, and the same amounts of protein were separated by 10% SDS-PAGE and blotted onto polyvinylidene fluoride membranes (Millipore, United States of America). Bands were visualized using chemiluminescence.

### 2.5 Immunohistochemistry

Immunohistochemistry analysis was performed as described previously (Yang et al., 2019; Du et al., 2021). Samples were fixed in 4% paraformaldehyde (PFA), paraffin-embedded and cut into 4–6 μm slides. All slides were dehydrated in gradient ethanol. Following the antigen retrieval done in 10 mM citrate buffer pH 6.0 (Na3H6H5O7, Beyotime, China), blocking was done using PBS with 10% normal goat serum (Sigma-Aldrich, United States of America) for 40 min. Slides were incubated overnight at 4°C with primary antibodies of IDO1 (ab211017, Abcam) or Ki67 (sc-23900, Santa Cruz). Slides were then incubated with goat anti-rabbit secondary antibody (G-21234, Thermo Fisher Scientific) or goat anti-mouse secondary antibody (G-21040, Thermo Fisher Scientific), stained using 3,3-diaminobenzidine solution and counterstained with hematoxylin.

### 2.6 Enzyme-linked immunosorbent assay (ELISA) analysis

The concentrations of Trp and Kyn in cell supernatant were detected with Elisa kits following the instructions of the manufacturer (MM-85226O2 and MM-926267O2, Meimian Biotech). The optical density was measured with a microplate reader (Thermo Fisher Scientific) at a wavelength of 450 nm. Concentrations were calculated by reference to the standard curve.

### 2.7 Exosome isolation and sequencing library preparation

Exosomes were isolated from cell culture supernatant with the exoEasy Maxi Kit (Qiagen, No. 76046, United States) according to the manufacturer’s instructions. Total RNA was isolated from exosomes following the protocol of the exoRNeasy SerumKit (Qiagen, No. 77064, United States). The RNA yield and exosome size range were analyzed on an Agilent 2,100 Bioanalyzer. Extracted RNA was used to prepare the miRNA sequencing library with QIAseq miRNA Library Kit (Qiagen, No. 331505, United States). The sequencing was performed on an Illumina sequencer at the Cloudseq Biotechnology Co., Ltd. (Shanghai, China).

### 2.8 Screening for differentially expressed miRNA and prediction of targets

Raw data were generated after sequencing, image analysis, base calling, and quality filtering on the Illumina sequencer. Firstly, Q30 was used to perform quality control. The adaptor sequences were trimmed and the adaptor-trimmed reads ( ≥ 15 nt) were left by cutadapt software (v1.9.3). Then, trimmed reads from all samples were pooled, and miRDeep2 software (v2.0.0.5) was used to predict novel miRNAs. The trimmed reads were aligned to the merged pre-miRNA databases (known pre-miRNA from miRBase v22 plus the newly predicted pre-miRNAs) using Novoalign software (v3.02.12) with at most one mismatch. The numbers of mature miRNA mapped tags were defined as the raw expression levels of that miRNA. The read counts were normalized by the edgeR approach. Differentially expressed miRNAs between two samples were filtered through Fold change. Differentially expressed miRNA between two groups was filtered by Fold Change and *p*-value. miRNA targets were performed based on Miranda and Targetscan, miRNA-targets networks were plotted by Cytoscape software (v2.8.0), and the GO analysis was performed based on the top 10 differentially expressed miRNA target genes.

### 2.9 Quantitative real-time PCR (qPCR)

Total RNA was isolated using Trizol (Invitrogen, CA, United States) following the manufacturer’s guidelines. Reverse transcription of mRNA was performed as previously described (Yang et al., 2019). Total RNAs of miRNA were reverse-transcribed to cDNA by using an All-in-one™ miRNA First-Strand cDNA Synthesis Kit (GeneCopoeia, Rockville, MD, United States) and Oligo (dT) priming method (Prime Script TMRT Reagent Kit; TaKaRa, Shiga, Japan). QPCR (Applied Biosystems 7,500, Foster City, CA, United States) was performed using power SYBR^®^ Green PCR Master Mix (Applied Biosystems). The expression levels of miRNAs and mRNAs were normalized to the levels of miR-103a-3p and β-Actin, respectively. qPCR analysis was performed in quadruplicate for each sample with specific primers: IDO1 Forward: 5′-ATG​CAA​GAA​CGG​GAC​ACT-3′, Reverse: 5′-GCC​TTT​CCA​GCC​AGA​CAA-3’; β-Actin Forward: 5′-GGG​AAA​TCG​TGC​GTG​AC-3′, Reverse: 5′-GGA​AGG​AAG​GCT​GGA​AGA​G-3’; hsa-miR-23a-3p Reverse primer: 5′-GTC​GTA​TCC​AGT​GCA​GGG​TCC​GAG​GTA​TTC​GCA​CTG​GAT​ACG​AC GG AAAT-3′, Forward: 5′-GCG​ATC​ACA​TTG​CCA​GGG-3′, Reverse: 5′- AGTGCAGGGTCCGAGGT ATT-3’; hsa-miR-103a-3p Reverse primer: 5′- GTC​GTA​TCC​AGT​GCA​GGG​TCC​GAG GTATTCG CAC​TGG​ATA​CGA​CTC​ATA​G-3′, Forward: 5′-GCG​AGC​AGC​ATT​GTA​CAG​GG-3′, Reverse: 5′-AGT​GCA​GGG​TCC​GAG​GTA​T T-3’.

### 2.10 Statistical analysis

All graphing and statistical analyses were performed using GraphPad Prism (version 9). All microscopy images were randomly taken from different areas. The positive count of each immunostaining photo ranged from 0 to 300 and was calculated as the percentage of weakly stained cells plus the percentage of moderately stained cells multiplied by two plus the percentage of strongly stained cells multiplied by three. Details of sample size n), statistical test, and *p*-value applied for each experiment were indicated in the figure legends. Results are presented as median ±SD or SEM. The statistical significance of differences between the two groups was assessed by Student’s t-test. One-way analysis of variance (ANOVA) was used to compare several treatment groups with one control group. *p* values <0.05 were considered statistically significant.

## 3 Results

### 3.1 IDO1 is highly expressed in tumor tissues and associates with the poor prognosis of OS patients

**FIGURE 1:**
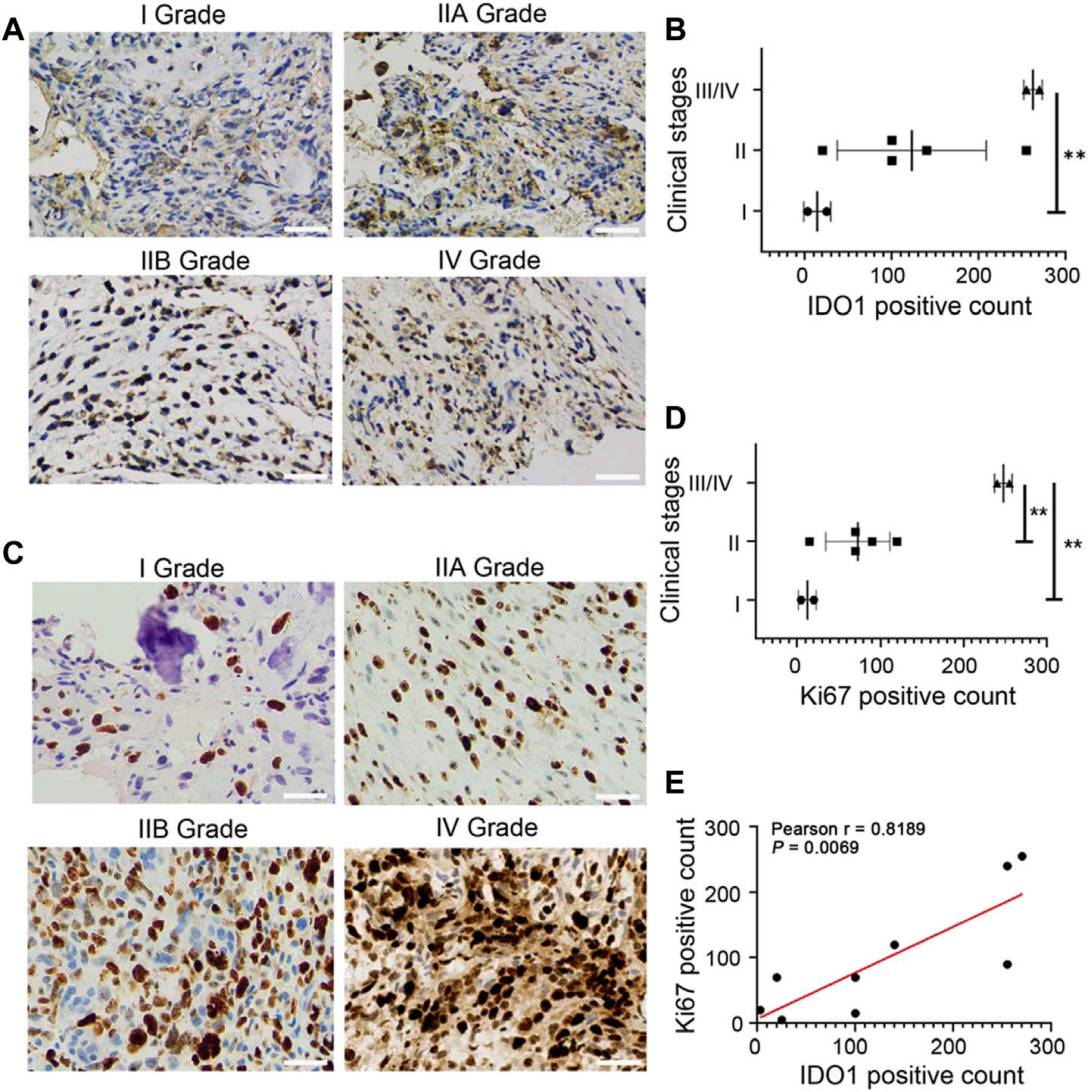
IDO1 is highly expressed in tumor tissues and associates with the poor prognosis of OS patients. **(A)** The representative immunostaining photos for IDO1 in tumor tissues of OS patients with different clinical stages. Magnification ×200. **(B)** The statistical graph of IDO1 expression in OS patients with different clinical stages. **(C)** The representative immunostaining photos for Ki67 in tumor tissues of OS patients with different clinical stages. Magnification ×400. **(D)** The statistical graph of Ki67 expression in OS patients with different clinical stages. Grade I, *n* = 2; Grade IIA/IIB, n = 5; Grade III/IV, *n* = 2. **(E)** Correlation between IDO1 positive cells and Ki67 positive cells in OS tissues. Statistical significance was determined by Student’s t-test and one-way ANOVA followed by Dunnett’s test. *p < 0.05, **p < 0.01, bars show the group mean ± SEM.

Among 9 cases of OS tissues, 66.7% (6/9) showed moderately or strongly positive immunohistochemical staining signal of IDO1, and 33.3% (3/9) weakly positive (Figure 1A). The IDO1 positive count of each immunostaining photo was calculated, and the relationship between IDO1 positive count and clinical stage of the patient was analysed. It was found that in II and III/IV grade OS patients the expression of IDO1 was significantly higher than that in I grade (Figure 1B). , We then performed Ki67 immunostaining on tumor tissue and obtained the Ki67 positive count of patients, since a higher Ki67 immunostaining is associated with a higher grade of malignancies and poorer prognosis of patients (Mardanpour et al., 2016; Kim et al., 2021). Ki67, like IDO1, is differently-expressed in OS tissues (Figure 1C), and the Ki67 index was positively correlated with the clinical grade of OS patients (Figures 1D,E). Based on the above results, it was deduced that the expression of IDO1 were involved in the malignancy of OS.

### 3.2 Higher IDO1 level is associated with poorer prognostic related clinical features of OS

**FIGURE 2:**
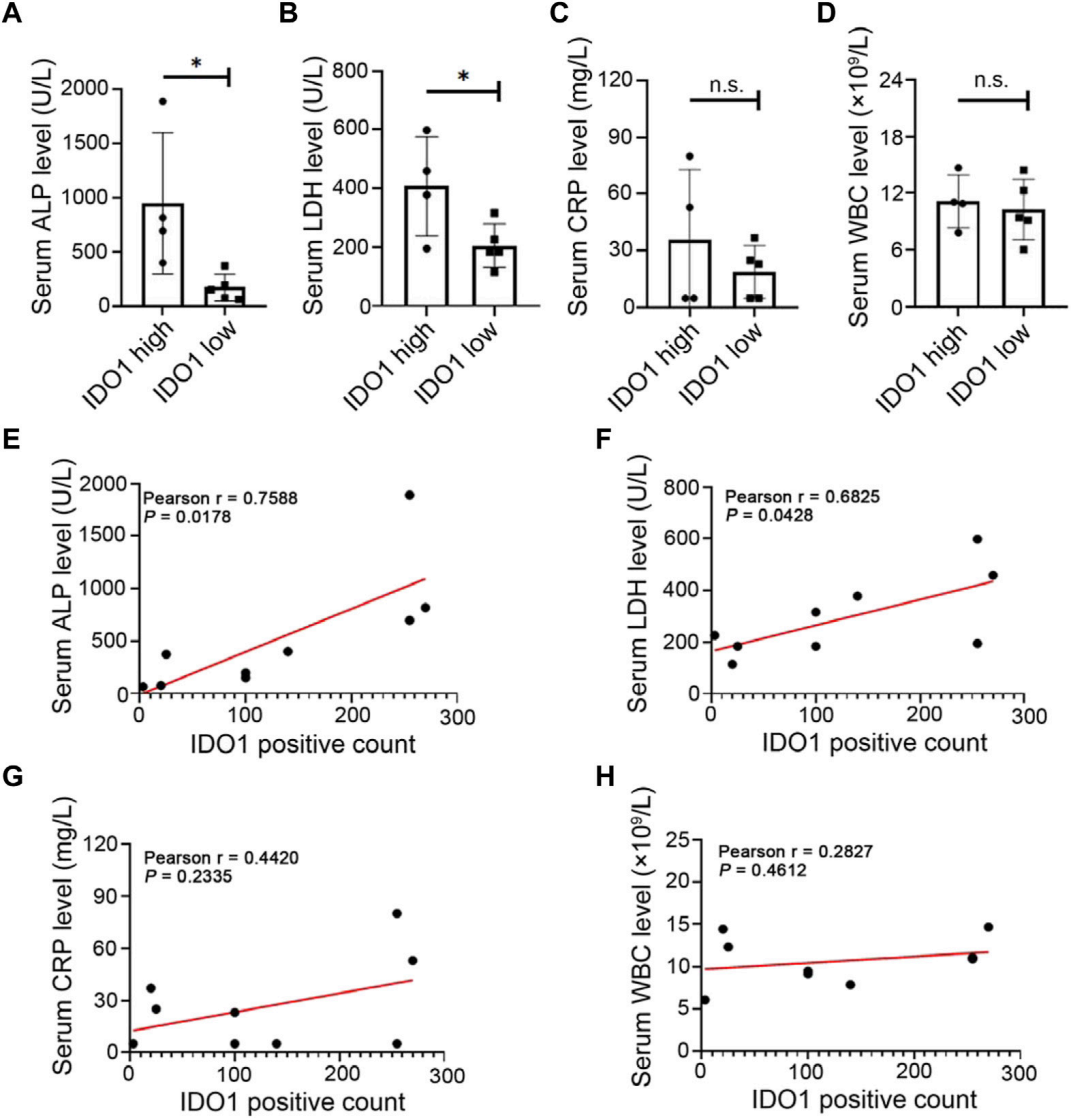
Upregulation of IDO1 is associated with prognostic related clinical features of OS. **(A–D)** The level of laboratory test indexes in OS patients with high or low expression of IDO1. Laboratory test indexes including serum ALP, LDH, CRP and WBC at diagnosis of OS patients. **(E–H)** Correlation between IDO1 expression and serum level of ALP, LDH, CRP or WBC. N = 9, including IDO1-high group, *n* = 4; IDO1-low group, *n* = 5. Statistical significance was determined by Student’s t-test and one-way ANOVA followed by Dunnett’s test. *p < 0.05, n. s., no significant difference, bars show the group mean ± SEM.

To investigate the correlation between IDO1 expression and prognostic related laboratory test indexes, patients were divided into IDO1-high or IDO1-low group according to the average positive count of IDO1 in their tumor tissues. Additionally, laboratory test indexes at diagnosis of these OS patients were collected (Table 1), including serum alkaline phosphatase (ALP) (Hao et al., 2017; Bao et al., 2019), lactate dehydrogenase (LDH) (Fu et al., 2018), white blood cell (WBC) count (Viola et al., 2021) and C-reactive protein (CRP) (Jettoo et al., 2019), as they are prognostic factors for OS reported in multiple studies. We found that between IDO1-high and IDO1-low groups, serum ALP and LDH concentrations showed significant difference, while WBC and CRP level did not (Figures 2A–D). Moreover, serum ALP and LDH concentrations but not WBC and CRP were significantly positively correlated with IDO1 expression (Figures 2E–H). These results indicated that IDO1 expression was positively correlated with the poor prognosis of patients with OS.

**TABLE 1:** Correlation between IDO1 expression and laboratory test indexes in patients with osteosarcoma.

Parameters	Description	No of patient	Ido1 positive count (mean)		*p*-value
			High	Low	
ALP	≤ 297	4	951.500	173.600	0.0324^✱^
(U/L)	> 297	5			
LDH	≤ 290	5	407.500	205.000	0.0444^✱^
(U/L)	> 290	4			
CRP	≤ 5	4	35.750	19.000	0.0916
(mg/L)	> 5	5			
WBC	≤ 10	4	11.138	10.290	0.3391
(×10^9^/L)	> 10	5			

### 3.3 IDO1 overexpression significantly affects exosomal miRNA subsets derived from osteosarcoma and ordinary osteoblasts

**FIGURE 3:**
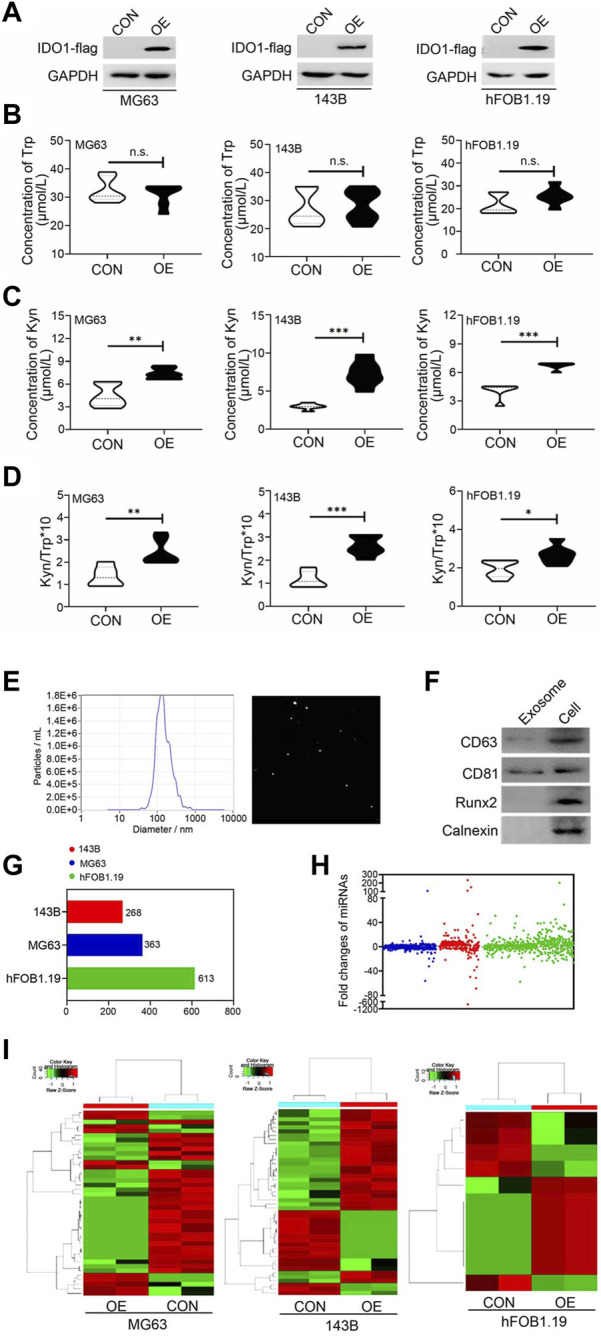
IDO1 overexpression significantly affects exosomal miRNA subsets derived from osteosarcoma and ordinary osteoblasts. **(A)** Western blot analysis of the IDO1 expression in IDO1 stably overexpressed cells and control cells. CON: exosomes isolated from cells that transfected with lenti-GFP-NC; OE: exosomes isolated from cells that transfected with lenti-GFP-IDO1. **(B–D)** Elisa analysis of Trp and Kyn levels and the Kyn/Trp ratio in the cell supernatant. **(E)** The analysis of diameter sizes and concentrations of isolated exosomes by nanoparticle tracking analysis device. **(F)** Western blot analysis of the exosomal markers in cell total protein sample (Cell) and isolated exosomes (Exosome). **(G, H)** Next-generation sequencing analysis of the type and number of miRNAs in exosomes of different cells. **(I)** The hierarchical cluster analysis of expression profile of miRNAs. Statistical significance was determined by Student’s t-test and one-way ANOVA followed by Dunnett’s test. **p < 0.01, ***p < 0.001, no significant difference, bars show the group mean ± SEM.

Dysregulation of exosomal miRNAs has been reported to be associated with the malignancy of OS (Vickers et al., 2011; Redis et al., 2012). To investigate whether IDO1 participated in OS malignant progression through miRNA, we selected non-metastatic OS cell line (MG63), metastatic OS cell line (143B) and non-tumorigenic immortalized osteoblastic hFOB1.19 cell line as the research object (Ren et al., 2015; Jerez et al., 2019). IDO1-overexpressing, stably transfected cell lines of these cells (MG63 OE, 143B OE and hFOB1.19 OE) were constructed and validated as IDO1 expression were significantly higher in these cells than that of their corresponding control cells (MG63 CON, 143B CON and hFOB1.19 CON) (Figure 3A). IDO1 activity was represented by the concentration of Kyn and the ratio of Kyn to Trp in cell supernatant. We found that the concentration of Trp in the supernatant of MG63 OE, 143B OE and hFOB1.19 OE cells was not significantly different from that of corresponding control cells (Figure 3B). However, the concentration of Kyn and the ratio of Kyn/Trp of these OE cells were significantly higher (Figures 3C,D).

Exosomes were isolated from conditioned culture media of these cells. The diameter sizes and concentrations of isolated exosomes were analyzed by Zetaview nanoparticle tracking analyzer. The peak diameter of the most of exosomes is 125 nm (Figure 3E), which is consistent with the report in the literature (Jerez et al., 2019). These exosomes also expressed characteristic exosomal markers (Figure 3F), indicating successful extraction. Next-generation sequencing was conducted to identify miRNAs enriched in exosomes in our panel of cell lines. A total of 1,244 miRNAs were sequenced and identified (Figures 3G,H), in which fold changes>2.0 and *p*-value < 0.05 were used as the screening threshold for differently expressed miRNAs. The expression profile of miRNAs was distinguishable in hierarchical cluster analysis (Figure 3I). Taken together, the detection of cell-specific miRNAs in these cell lines indicates considerable heterogeneity in the presence of miRNAs in exosomes, not only consistent with the distinct biological phenotypes of each cell type but also affected by the expression of IDO1.

### 3.4 IDO1 maintains the malignancy of osteosarcoma cells by upregulating hsa-miR-23a-3p in exosomes

**FIGURE 4:**
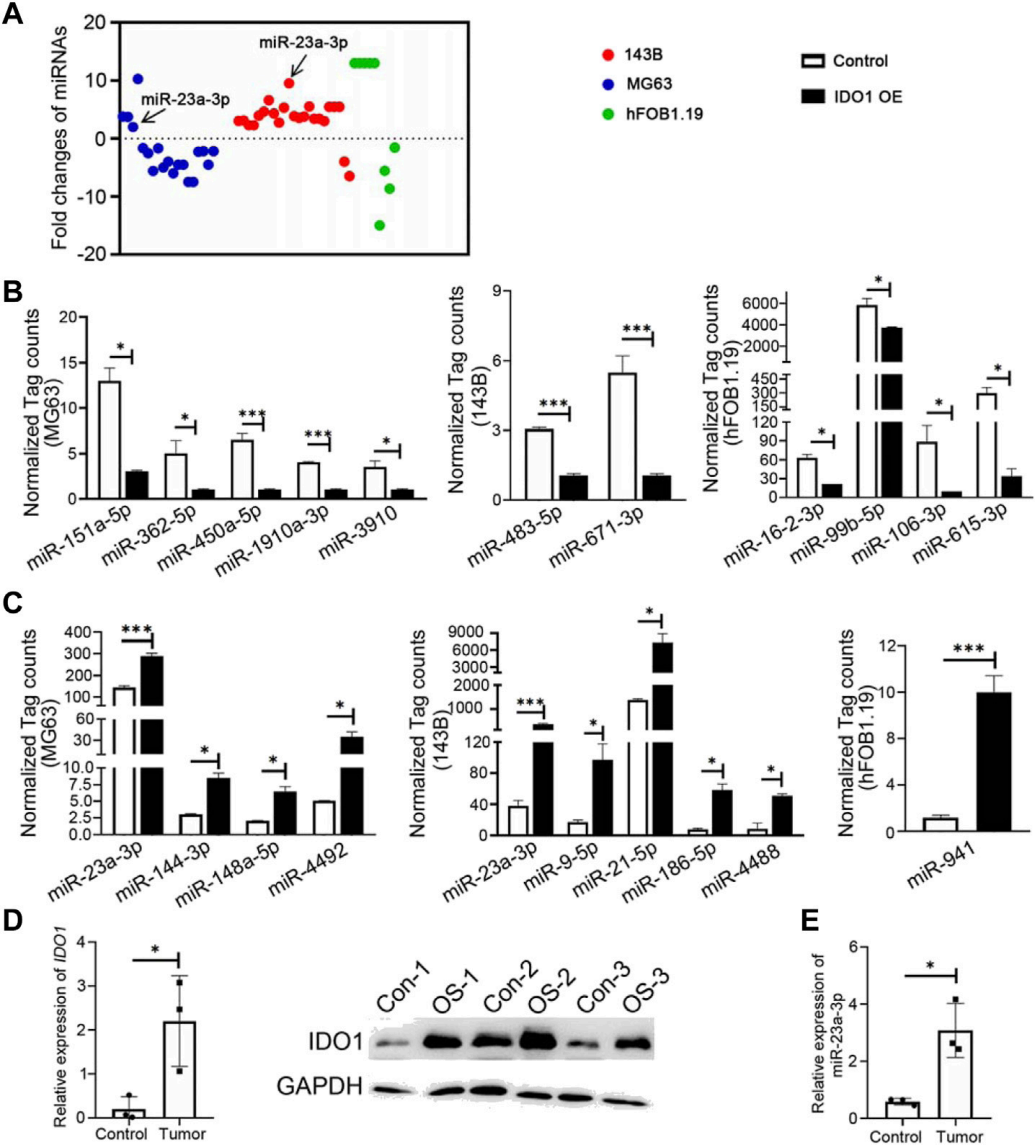
IDO1 maintains the characteristics of malignant osteosarcoma cells by upregulating hsa-miR-23a-3p in exosomes. **(A)** Identification of differently expressed miRNAs in 3 cell groups of MG63, 143B, and hFOB1.19. **(B, C)** The normalized tag count analysis of top significantly downregulated and upregulated miRNAs in MG63 OE, 143B OE, and hFOB1.19 OE cells and their control cells. Control: Exosomes isolated from cells that transfected with lenti-GFP-NC; IDO1 OE: Exosomes isolated from cells that transfected with lenti-GFP-IDO1. **(D)** The analysis of mRNA and protein expressions of IDO1 in clinical specimens by qPCR and Western blot. **(E)** The analysis of mRNA expressions of hsa-miR-23a-3p in clinical specimens by qPCR. N = 3. Tumor/OS-1,2,3: tumor tissues of osteosarcoma patients; Control/CON-1,2,3: bone tissues of multi-fingered patients. Statistical significance was determined by Student’s t-test and one-way ANOVA followed by Dunnett’s test. *p < 0.05, **p < 0.01, ***p < 0.001, bars show the group mean ± SEM.

96 differently expressed miRNAs (DE miRNAs) were identified in 3 cell groups by comparing MG63 OE, 143B OE and hFOB1.19 OE with their control cells. We found that hsa-miR-23a-3p was only significantly upregulated in both MG63 and 143B OS cells but not non-tumorigenic immortalized osteoblastic hFOB1.19 cells (Figure 4A). To confirm this result, we further screened for top DE miRNAs in the above three groups of cells. Nine top significantly upregulated and downregulated miRNAs were detected from MG63 cells (Table 2), 7 from 143B cells (Table 3), and 5 from hFoB1.19 cells (Table 4). We found that compared with non-tumorigenic immortalized osteoblastic hFOB1.19 cells, MG63 and 143B OS cells did not share the same downregulated exosomal miRNAs, and only has-miR-23a-3p was significantly upregulated in both OS cells (Figures 4B,C).

**TABLE 2:** The top 9 up- and downregulated miRNAs in MG63 cells.

DE miRNAs	Regulation	Log2 (fold change)	*p*-value	Sequence
hsa-miR-23a-3p	up	1.99	0.0062	AUC​ACA​UUG​CCA​GGG​AUU​UCC
hsa-miR-144-3p	up	3.80	0.047	UAC​AGU​AUA​GAU​GAU​GUA​CU
hsa-miR-148a-5p	up	3.70	0.039	AAA​GUU​CUG​AGA​CAC​UCC​GAC​U
hsa-miR-4492	up	10.29	0.028	GGGGCUGGGCGCGCGCC
hsa-miR-151a-5p	down	−5.60	0.024	UCG​AGG​AGC​UCA​CAG​UCU​AGU
hsa-miR-362-5p	down	−6.00	0.038	AAU​CCU​UGG​AAC​CUA​GGU​GUG​AGU
hsa-miR-450a-5p	down	−7.50	0.0059	UUU​UGC​GAU​GUG​UUC​CUA​AUA​U
hsa-miR-1910-3p	down	−5.00	0.00010	GAG​GCA​GAA​GCA​GGA​UGA​CA
hsa-miR-3910	down	−4.50	0.020	AAA​GGC​AUA​AAA​CCA​AGA​CA

**TABLE 3:** The top 7 up- and downregulated miRNAs in 143B cells.

DE miRNAs	Regulation	Log2 (fold change)	*p*-value	Sequence
hsa-miR-23a-3p	up	9.53	0.0033	AUC​ACA​UUG​CCA​GGG​AUU​UCC
hsa-miR-9-5p	up	5.47	0.032	UCU​UUG​GUU​AUC​UAG​CUG​UAU​GA
hsa-miR-21-5p	up	5.32	0.033	UAG​CUU​AUC​AGA​CUG​AUG​UUG​A
hsa-miR-186-5p	up	6.11	0.012	CAA​AGA​AUU​CUC​CUU​UUG​GGC​U
hsa-miR-4488	up	5.53	0.017	AGGGGGCGGGCUCCGGCG
hsa-miR-483-5p	down	−4.00	0.00010	AAG​ACG​GGA​GGA​AAG​AAG​GGA​G
hsa-miR-671-3p	down	−6.50	0.0082	UCC​GGU​UCU​CAG​GGC​UCC​ACC

**TABLE 4:** The top 5 up- and downregulated miRNAs in hFoB1.19 cells.

DE miRNAs	Regulation	Log2 (fold change)	*p*-value	Sequence
hsa-miR-941	up	13.00	0.0069	CAC​CCG​GCU​GUG​UGC​ACA​UGU​GC
hsa-miR-16-2-3p	down	−5.57	0.043	CCA​AUA​UUA​CUG​UGC​UGC​UUU​A
hsa-miR-99b-5p	down	−1.57	0.039	CAC​CCG​UAG​AAC​CGA​CCU​UGC​G
hsa-miR-106b-3p	down	−15.00	0.046	CCG​CAC​UGU​GGG​UAC​UUG​CUG​C
hsa-miR-615-3p	down	−8.67	0.025	UCC​GAG​CCU​GGG​UCU​CCC​UCU​U

We also verified the above results in clinical samples. The mRNA and protein expressions of IDO1 in OS tumor tissues were significantly higher than those in normal bone tissues excised from control multi fingered patients (Figure 4D). It was found that the expression of hsa-miR-23a-3p was higher in tumor tissues than that in control tissues (Figure 4E). MiRNAs in cancer cells act as post-transcriptional regulators of their mRNA targets to control the expression of genes involved in tumor progression (Nugent, 2014). To understand the biological functions of has-miR-23a-3p, we performed biological processes and cell components analysis by GO enrichment analysis using the protein interaction network database (string-db.com) (Szklarczyk et al., 2019) (Table 5). As the data suggests, has-miR-23a-3p targets include genes in fatty acid metabolic pathway and muscle system process which have been report to be a typical sign during cancer development (Hoy et al., 2021) (Supplementary Material). Therefore, upregulated hsa-miR-23a-3p in IDO1 overexpressed OS cell lines may signal other cells through exosomes to shape the tumor microenvironment towards tumor survival. In summary, we believe that hsa-miR-23a-3p may be the key factor for OS cells with high IDO1 level to maintain its malignancy.

**TABLE 5:** GO enrichment analysis of hsa-miR-23a-3p targets using protein interaction network database.

Go term ID	GO term description	Target genes
GO:0003012	Muscle system process	MYH1, MYH4, NCOA6, PPARGC1A, PRDM10, TRIM63
GO:0014878	Response to electrical stimulus involved in regulation of muscle adaptation	
GO:0014732	Skeletal muscle atrophy	
GO:0021877	Forebrain neuron fate commitment	EFHC2, EOMES, FOXP2, MESP1, MYOCD, NKX2-1, POU4F2, SATB2, TXK, UBL3
GO:0010002	Cardioblast differentiation	
GO:0035886	Vascular associated smooth muscle cell differentiation	
GO:0051952	Regulation of amine transport	A1BG, ADRA2A, CBLN1, CCK, CNR1, CXCL12, GABRG1, MET, NTS, RGS8, STAT5B
GO:0007186	G protein-coupled receptor signaling pathway	
GO:0050433	Regulation of catecholamine secretion	
GO:0006635	Fatty acid beta-oxidation	ACAA1, ACSBG1, ASAH2, ATP5A1, ATP5L, AUH, BET1, ECHDC1, FAM114A2, FASTKD3, FRMD5, GPR22, HADHB, LIPF, LMAN1, MOGS, NDUFA2, OXCT1, PCDH18, RBM47, RBPMS2, SGPP1, SLC25A17, TMED5, UQCRFS1
GO:0006631	Fatty acid metabolic process	
GO:0044242	Cellular lipid catabolic process	
GO:0031461	cullin-RING ubiquitin ligase complex	COMMD6, CRBN, CUL3, CUL4A, ENC1, KLHL13, NEMF, PELO, RIPK4, RPLP1, SIKE1, STRIP1
GO:0031463	Cul3-RING ubiquitin ligase complex	
GO:0031464	Cul4A-RING E3 ubiquitin ligase complex	

### 3.5 IDO1 promotes the immunosuppressive properties of osteosarcoma cells by regulating the expression of differently expressed miRNA between 143B and MG63 cells

**FIGURE 5:**
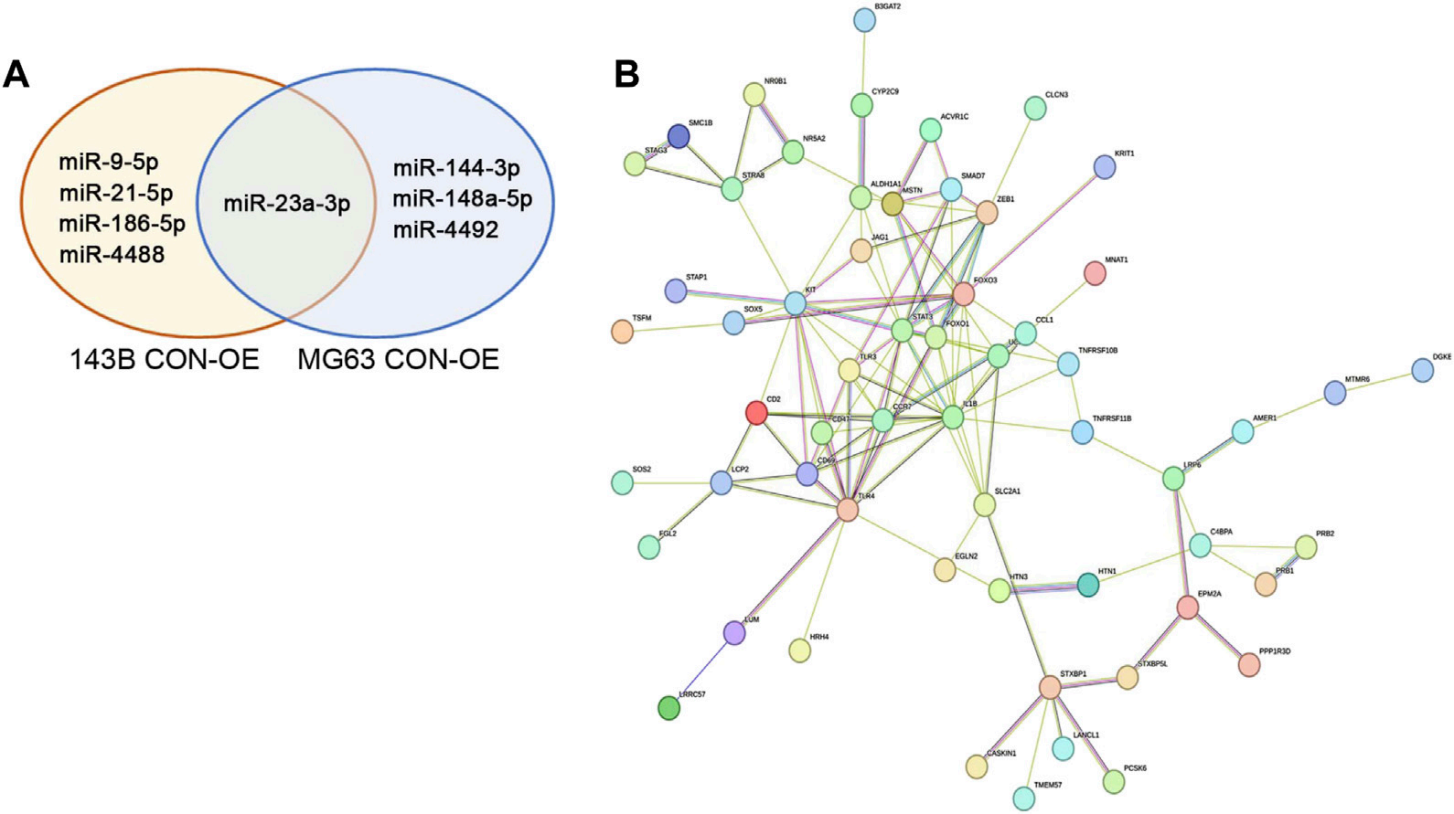
IDO1 promotes the immunosuppressive properties of osteosarcoma cells by regulating the expression of differently expressed miRNA between 143B and MG63 cells, including hsa-miR-21-5p and hsa-miR-4492. **(A)** Venn diagram depiction of significantly upregulated miRNA in 143B and MG63 cells. **(B)** The representative protein interaction network of hsa-miR-21a-5p target genes. CON: exosomes isolated from cells that transfected with lenti-GFP-NC; OE: exosomes isolated from cells that transfected with lenti-GFP-IDO1.

Since upregulation of hsa-miR-23a-3p is shared between the two OE cell lines we examined and thus believed to have similar role in these cells, we hypothesized that other differently expressed miRNAs which are not shared between 143B OE and MG63 OE cells may contribute to their unique properties different from each other. We then investigated the function of these miRNAs using the same method and databases as mentioned above (Figure 5A; Supplementary Material). We found that hsa-miR-21-5p, hsa-miR-9-5p, hsa-miR-4488 and hsa-miR-186-5p were upregulated in 143B OE cells but not in MG63 OE cells. Many target genes of above-mentioned miRNAs showed no significant GO enrichment, while targets of hsa-miR-21-5p yielded interesting results (Supplementary Material). Among the target genes, Signal transducer and activator of transcription 3 (*STAT3*), transcription factor forkhead box O1 (*FOXO1*), Toll-like receptor 4 (*TLR4*) and transcription factor forkhead box O3 (*FOXO3*), which are involved in immune regulation and tumor cell growth and metabolism, are the most closely connected (Figure 5B). GO analysis of targets hsa-miR-21-5p has shown enrichment in “positive regulation of cytokines production involved in inflammatory response” as well as “interleukin-1 beta production” and could involve in immunosuppressive action (Supplementary Material).

Similarly, the target genes of hsa-miR-4492, hsa-miR-144-3p, and hsa-miR-148a-5p, upregulated miRNAs in MG63 OE cells but not in 143B OE cells were also analyzed (Figure 5A; Supplementary Material). We discovered that target genes of hsa-miR-4492 were mostly grouped towards immune related functions, such as basophil differentiation, dendritic cell differentiation, and positive regulation of T-helper 2 cell cytokine production (Supplementary Material), suggesting its immune suppression role in tumor microenvironment, since it could downregulate said genes in host cells. These results indicate that both cell lines have the ability of employing different strategy to evade host immune response with their own unique miRNAs, and to assure tumor survival.

## 4 Discussion

OS is one of the most common malignant tumors that pose serious threat to adolescent health (Morrow and Khanna, 2015; Kager and Bielack, 2017; Taran et al., 2017). Although many attempts and efforts have been made in the immunotherapy of OS, the clinical trial results are still not optimistic (Wedekind et al., 2018). Therefore, developing new effective treatment strategies and targets remains the focus of OS immunotherapy research. IDO1 is one of the key targets of tumor immunotherapy, but the research on its role in OS progression is still in the initial stage. Although there are no previous reports suggesting that IDO1 may shape the cancer immune landscape in the TME and promote tumor progression by OS-derived exosome miRNAs, the significance of IDO1 in OS has been described earlier (Toda et al., 2020). In addition, the roles of various miRNAs in the progression of OS have also been report previously (Wang et al., 2019). We believe our study may provide a bridging between these previously independent topics.

To investigate the role of IDO1 in the progression of OS, we analyzed the expression of IDO1 in tumor tissues of OS patients and found that IDO1 is differently expressed in tumor tissues and associated with the poor prognosis of OS patients. Tumor cells upregulates IDO1 expression and induce immune tolerance in the tumor microenvironment by depleting the essential amino acid Trp of the substrate and generating toxic metabolites such as Kyn (Takikawa et al., 1986; Routy et al., 2016; Yang et al., 2019). Urakawa et al. investigated the expression of IDO1 and Foxp3 in tumor tissue samples of OS patients, and their expression was closely related to the poor prognosis of patients (Urakawa et al., 2009; Zheng and Wan, 2018). They proposed that IDO1 might be the target molecule of OS immunotherapy. Recently, the analysis of the OS database found that the immune checkpoint IDO1 and PD-1, PD-L1 and B7-H3 were expressed in the tumor samples, among which the expression of IDO1 and B7-H3 was significantly related to the poor prognosis of patients (McEachron et al., 2018). These studies support our results on the relationship between IDO1 expression and clinical staging. Additionally, IDO1 expression has a significant positive correlation with the expression of Ki67. Similar to Ki67, serum ALP, LDH, WBC and CRP are also important prognostic factors for patients with OS (Yin et al., 2018; Menendez et al., 2022). In the present study, laboratory test indexes at diagnosis of recruited OS patients were collected and analyzed. It was found that the concentrations of ALP and LDH, rather than WBC and CPR, positively correlated with high IDO1 expression significantly. These results suggest that the expression of IDO1 is involved in the malignant progression of OS.

This study also revealed the potential role of differential expression of miRNA in exosomes derived from IDO1 expressing OS. Exosomes and their cargo like miRNAs are associated with OS progression and metastasis (Sun et al., 2018; Li et al., 2021). Our study showed that a total of 1244 DE miRNAs were identified in three groups of cells by comparing MG63 OE, 143B OE, and hFOB1.19 OE with their corresponding control cells. Hsa-miR-23a-3p was the only one significantly upregulated DE miRNA in both MG63 and 143B OS cells but not non-tumorigenic immortalized osteoblastic hFOB1.19 cells, which is consistent with the previous report that lncRNA GAS5 directly suppresses the expression of hsa-miR-23a-3p and inhibits the malignant progression of OS through PTEN/PI3K/AKT pathway (Liu et al., 2020). In the aspect of bioinformatic analysis, pioneering researchers used the StarBase database to build a competing endogenous RNA (ceRNA) network and find a LAMTOR5-AS1/hsa-miR-23a-3p/TP63 regulatory axis which is involved in the malignant progress of OS (Han et al., 2022). Meanwhile in our study, GO enrichment analysis showed that the target of hsa-miR-23a-3p was mainly involved in fatty acid metabolism pathway and muscle system process, which was closely related to cancer development. In summary, OS cells with higher IDO1 expression maintain their malignancy by regulating the level of hsa-miR-23a-3p. A similar strategy has been earlier employed by Zhao and his colleagues when they investigated the effect of tumor-derived exosomal miRNA miR-934 on macrophages. Zhao’s team studied the miRNA as we did in this study by extracting exosomes and using bioinformatic tools, while they further investigated the mechanism by which miRNA is packaged into the exosome, which is also a question to be further studied in our case (Zhao et al., 2020).

In the present study, we found that hsa-miR-21-5p, hsa-miR-9-5p, hsa-miR-1488 and hsa-miR-186-5p were highly expressed in 143B OE cells but not in MG63 OE cells, while hsa-miR-144-3p, hsa-miR-148a-5p, hsa-miR-4492 and hsa-miR-483-5p highly expressed in MG63 OE cells but not in 143B OE cells. Among the target genes of these hsa-miRNAs, only the targets of hsa-miR-21-5p and hsa-miR-4492 have significant GO analysis results. *STAT3, FOXO1, TLR4* and *FOXO3* are target genes of hsa-miR-21-5p, and they are closely related to each other. STAT3 is a key transcription factor that mediates the progression of OS and can play a role through JAK/STS3 (Zhu et al., 2021) or VEGFR2/STAT3/BCL-2 pathways (Liu et al., 2017). FOXO1 acts as a tumor suppressor in OS suppressed tumor progression by downregulating the Wnt/β-catenin pathway (Guan et al., 2015). *TLR4* is also a tumor suppressor gene of OS. As an immune-related and EMT-related gene of OS, it was detected by analyzing UCSC Xena and GEO database. It is also verified in patient tissues that TLR4 was significantly lower in the tumor cells than in the normal bone (Liang et al., 2021). Yahiro and his colleague found that TLR4 activation by LPS increased CD8^+^ cells infiltrating into lung metastases and suppressed OS progression in the mouse model (Yahiro et al., 2020). CtBP1-p300-FOXO3a transcriptional complex negatively regulates FOXO3a levels and represses the expression of the pro-apoptotic regulators Bax and Bim in human OS cells, finally promoting the tumor progression (Li et al., 2019). These genes are the key targets of hsa-miR-21a-5p and play an important role in the immunosuppression and growth metastasis of OS. In addition, the main function of the hsa-miR-4492 target analyzed by GO is related to tumor immunity, such as basophil differentiation, dendritic cell differentiation, and positive regulation of T-helper 2 cell cytokine production. The target genes include *LCP1*, which regulates the expression of basophil marker CD69 (Wabnitz et al., 2007); *TREM2*, mediating dendritic cell maturation (Bouchon et al., 2001); and *IL17*, which is involved in the regulation of T helper cells including TH1, TH2 and TH17 (Iwasaki et al., 2015). However, since only bioinformatic analysis is performed on these DE miRNAs, further validation using cell lines or clinical sample is required for future studies in order to confirm our speculation.

As our study has shown, differently expressed exosomal miRNAs correlated to IDO1 up- or downregulation functions differently in TME. DE miRNAs corresponded to higher level of IDO1 favors tumor survival, progression, and immune escape. Therefore, we believe treatment targeting IDO1 or directly, these miRNAs, could regulate the gene expressions in surrounding cells, shaping TME against tumor progression.

## 5 Conclusion

In summary, immunosuppressive enzyme IDO1 is a key immunotherapeutic target for OS. IDO1 affects the progression of OS by up regulating the expression of hsa-miR-23a-3p in OS derived exosomes. Therefore, targeting IDO1-regulated hsa-miR-23a-3p expression might be a potential therapeutic strategy for OS treatment.

## Data Availability

The data presented in the study are deposited in the Gene Expression Omnibus, accession number GSE232452. The link is https://www.ncbi.nlm.nih.gov/geo/query/acc.cgi?acc=GSE232452.
