# Supplementary material for: Indoleamine 2,3-dioxygenase 1 promotes osteosarcoma progression by regulating tumor-derived exosomal miRNA hsa-miR-23a-3p
Source: Front Pharmacol. 2023 May 22;14:1194094. doi: 10.3389/fphar.2023.1194094 (PMC10239870; doi:10.3389/fphar.2023.1194094)
Supplement: Supplementary file 1 [file Presentation1.PPTX]

## Slide 1
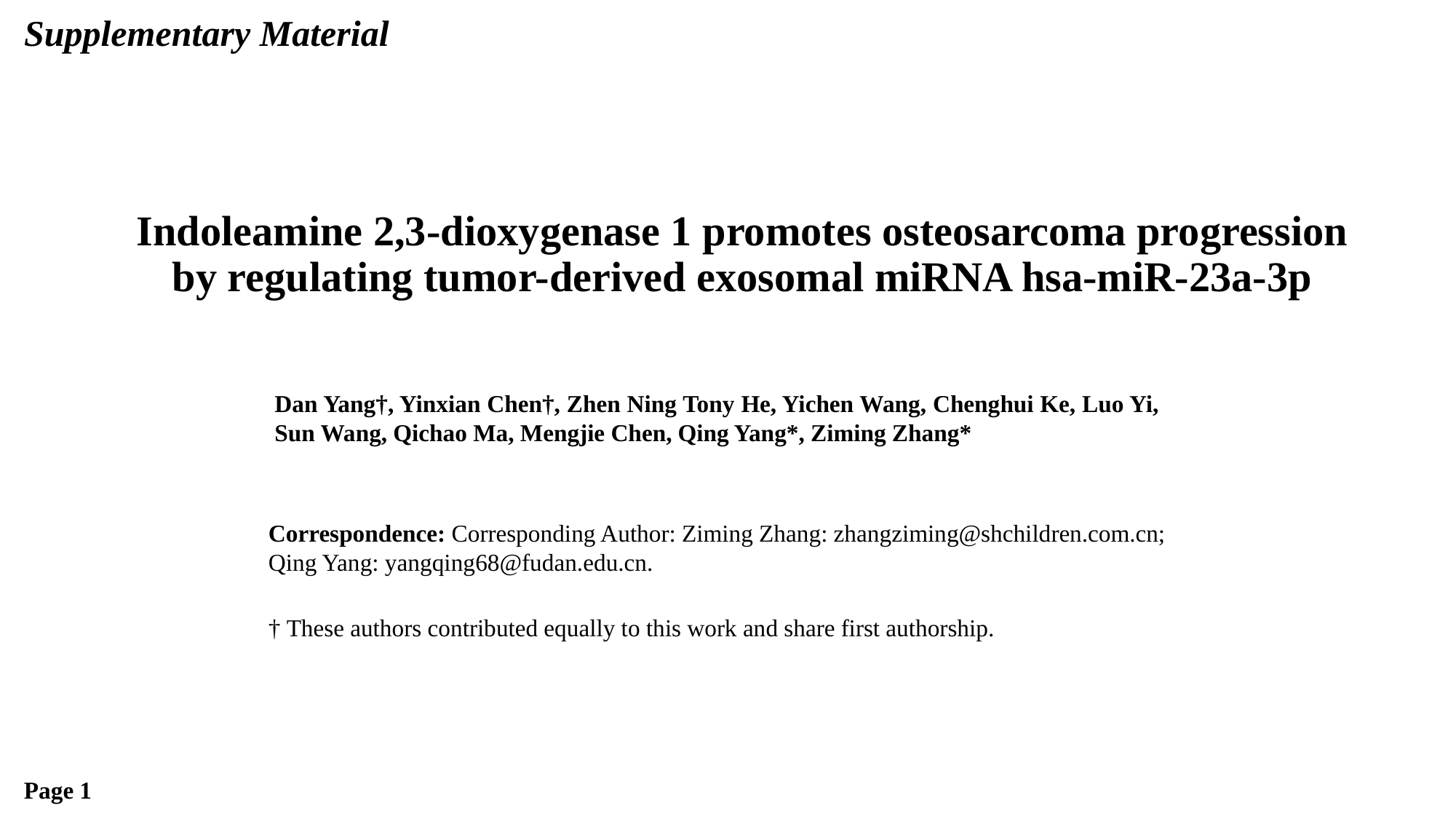

# Supplementary Material
Indoleamine 2,3-dioxygenase 1 promotes osteosarcoma progression by regulating tumor-derived exosomal miRNA hsa-miR-23a-3p
Dan Yang†, Yinxian Chen†, Zhen Ning Tony He, Yichen Wang, Chenghui Ke, Luo Yi, Sun Wang, Qichao Ma, Mengjie Chen, Qing Yang*, Ziming Zhang*
Correspondence: Corresponding Author: Ziming Zhang: zhangziming@shchildren.com.cn; Qing Yang: yangqing68@fudan.edu.cn.
† These authors contributed equally to this work and share first authorship.
Page 1

## Slide 2
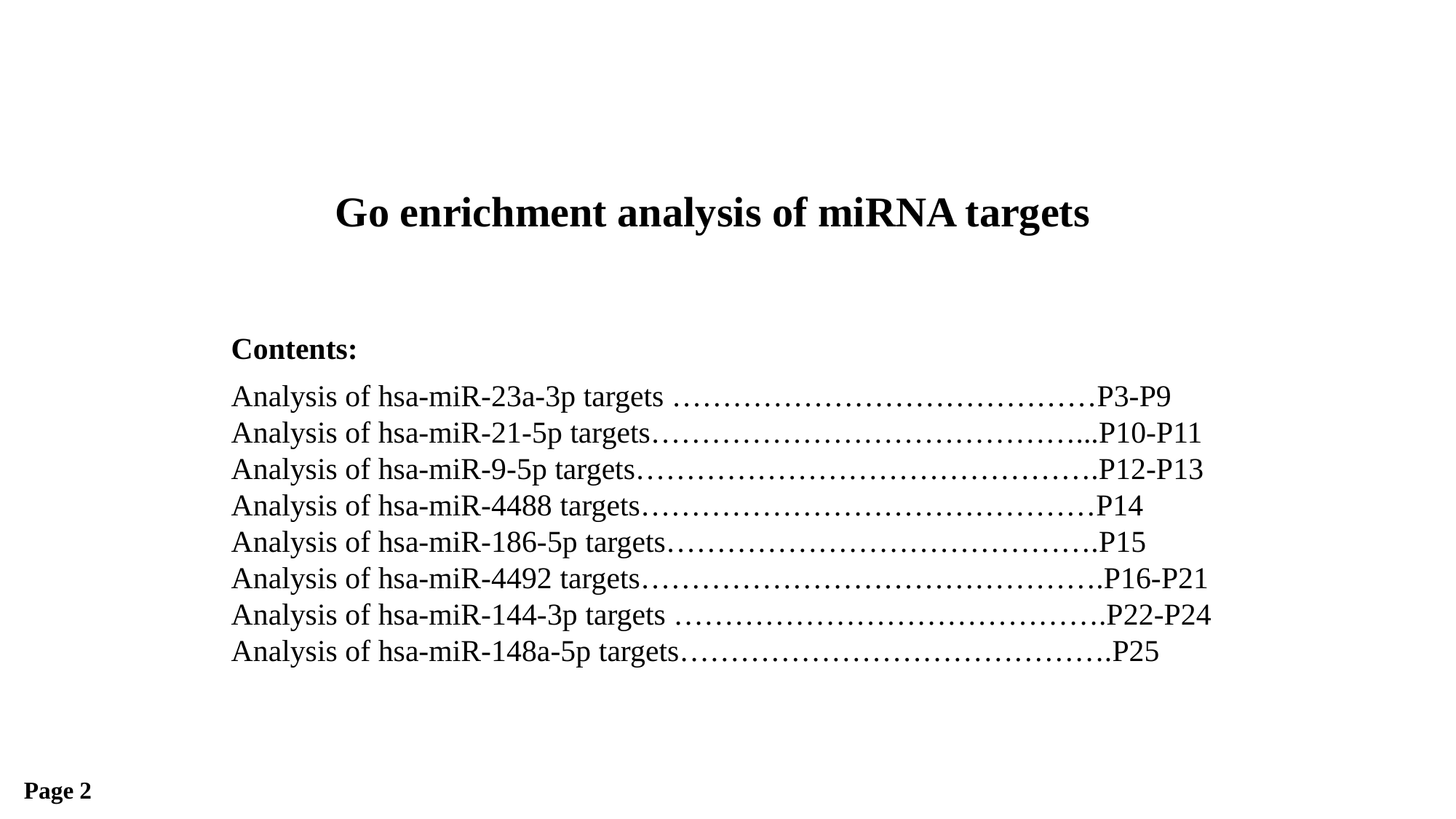

Go enrichment analysis of miRNA targets
Contents:
Analysis of hsa-miR-23a-3p targets ……………………………………P3-P9
Analysis of hsa-miR-21-5p targets……………………………………...P10-P11
Analysis of hsa-miR-9-5p targets……………………………………….P12-P13
Analysis of hsa-miR-4488 targets………………………………………P14
Analysis of hsa-miR-186-5p targets…………………………………….P15
Analysis of hsa-miR-4492 targets……………………………………….P16-P21
Analysis of hsa-miR-144-3p targets …………………………………….P22-P24
Analysis of hsa-miR-148a-5p targets…………………………………….P25
Page 2

## Slide 3
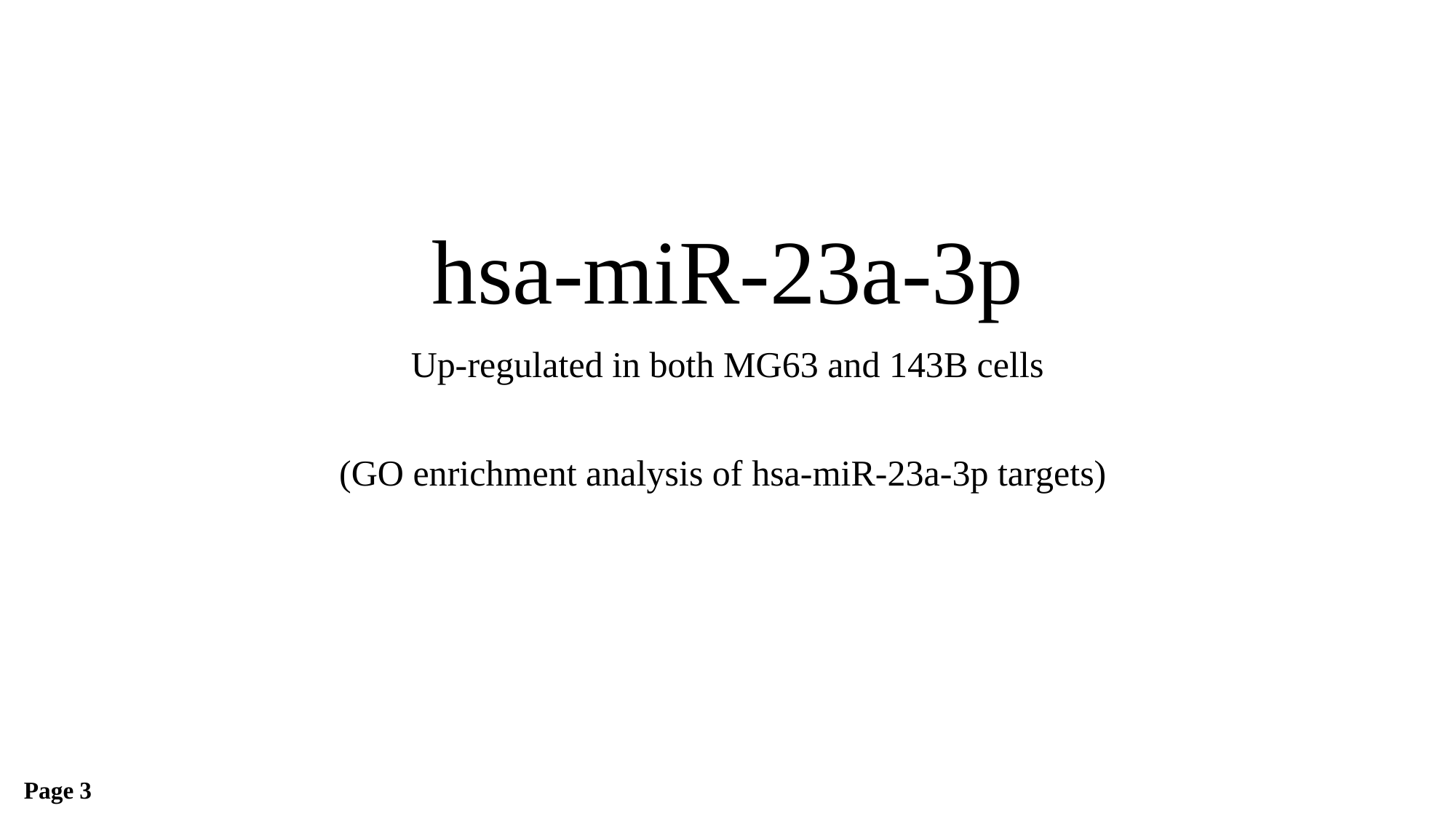

# hsa-miR-23a-3p
Up-regulated in both MG63 and 143B cells
(GO enrichment analysis of hsa-miR-23a-3p targets)
Page 3

## Slide 4
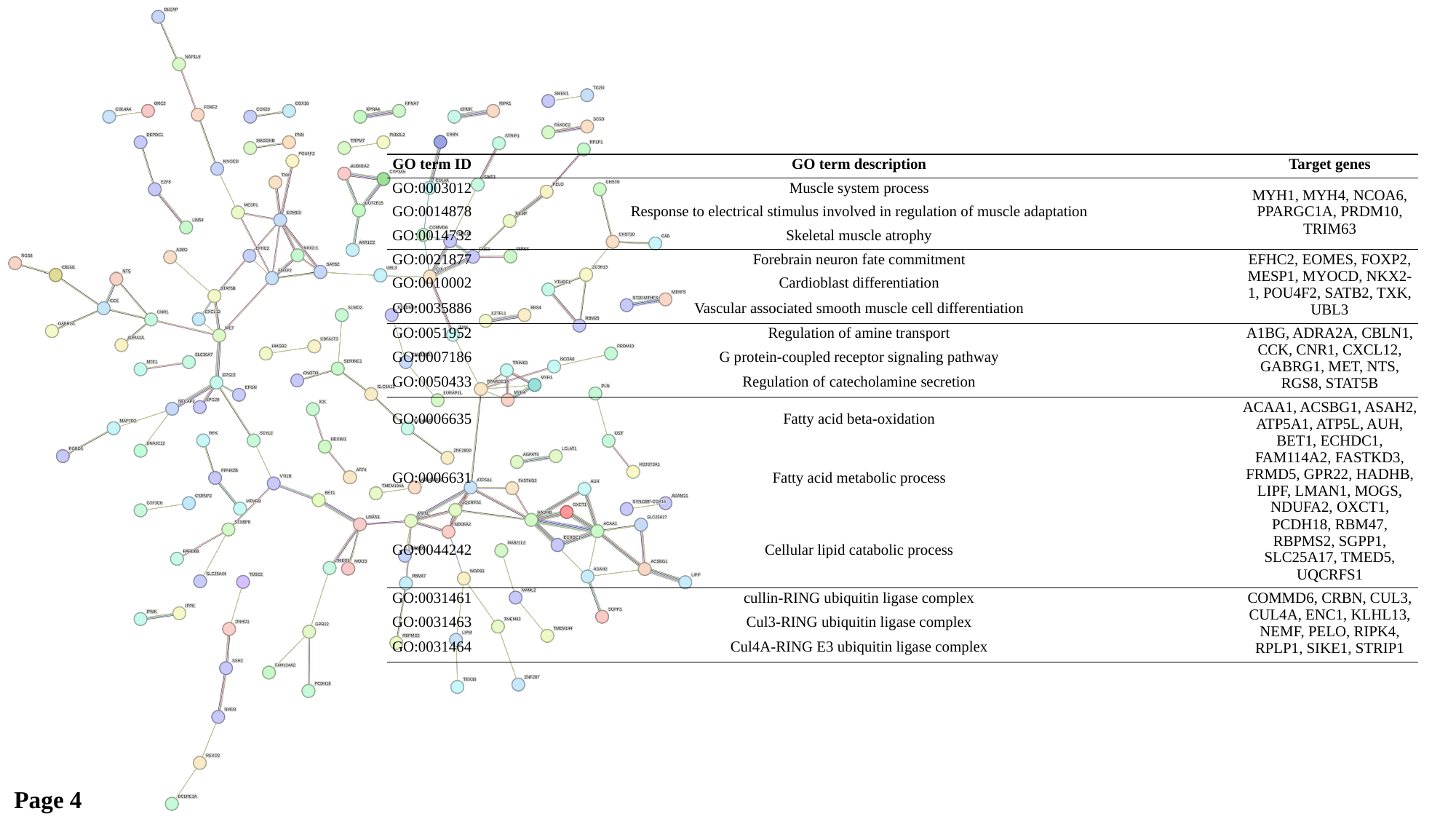

| GO term ID | GO term description | Target genes |
| --- | --- | --- |
| GO:0003012 | Muscle system process | MYH1, MYH4, NCOA6, PPARGC1A, PRDM10, TRIM63 |
| GO:0014878 | Response to electrical stimulus involved in regulation of muscle adaptation | |
| GO:0014732 | Skeletal muscle atrophy | |
| GO:0021877 | Forebrain neuron fate commitment | EFHC2, EOMES, FOXP2, MESP1, MYOCD, NKX2-1, POU4F2, SATB2, TXK, UBL3 |
| GO:0010002 | Cardioblast differentiation | |
| GO:0035886 | Vascular associated smooth muscle cell differentiation | |
| GO:0051952 | Regulation of amine transport | A1BG, ADRA2A, CBLN1, CCK, CNR1, CXCL12, GABRG1, MET, NTS, RGS8, STAT5B |
| GO:0007186 | G protein-coupled receptor signaling pathway | |
| GO:0050433 | Regulation of catecholamine secretion | |
| GO:0006635 | Fatty acid beta-oxidation | ACAA1, ACSBG1, ASAH2, ATP5A1, ATP5L, AUH, BET1, ECHDC1, FAM114A2, FASTKD3, FRMD5, GPR22, HADHB, LIPF, LMAN1, MOGS, NDUFA2, OXCT1, PCDH18, RBM47, RBPMS2, SGPP1, SLC25A17, TMED5, UQCRFS1 |
| GO:0006631 | Fatty acid metabolic process | |
| GO:0044242 | Cellular lipid catabolic process | |
| GO:0031461 | cullin-RING ubiquitin ligase complex | COMMD6, CRBN, CUL3, CUL4A, ENC1, KLHL13, NEMF, PELO, RIPK4, RPLP1, SIKE1, STRIP1 |
| GO:0031463 | Cul3-RING ubiquitin ligase complex | |
| GO:0031464 | Cul4A-RING E3 ubiquitin ligase complex | |
Page 4

## Slide 5
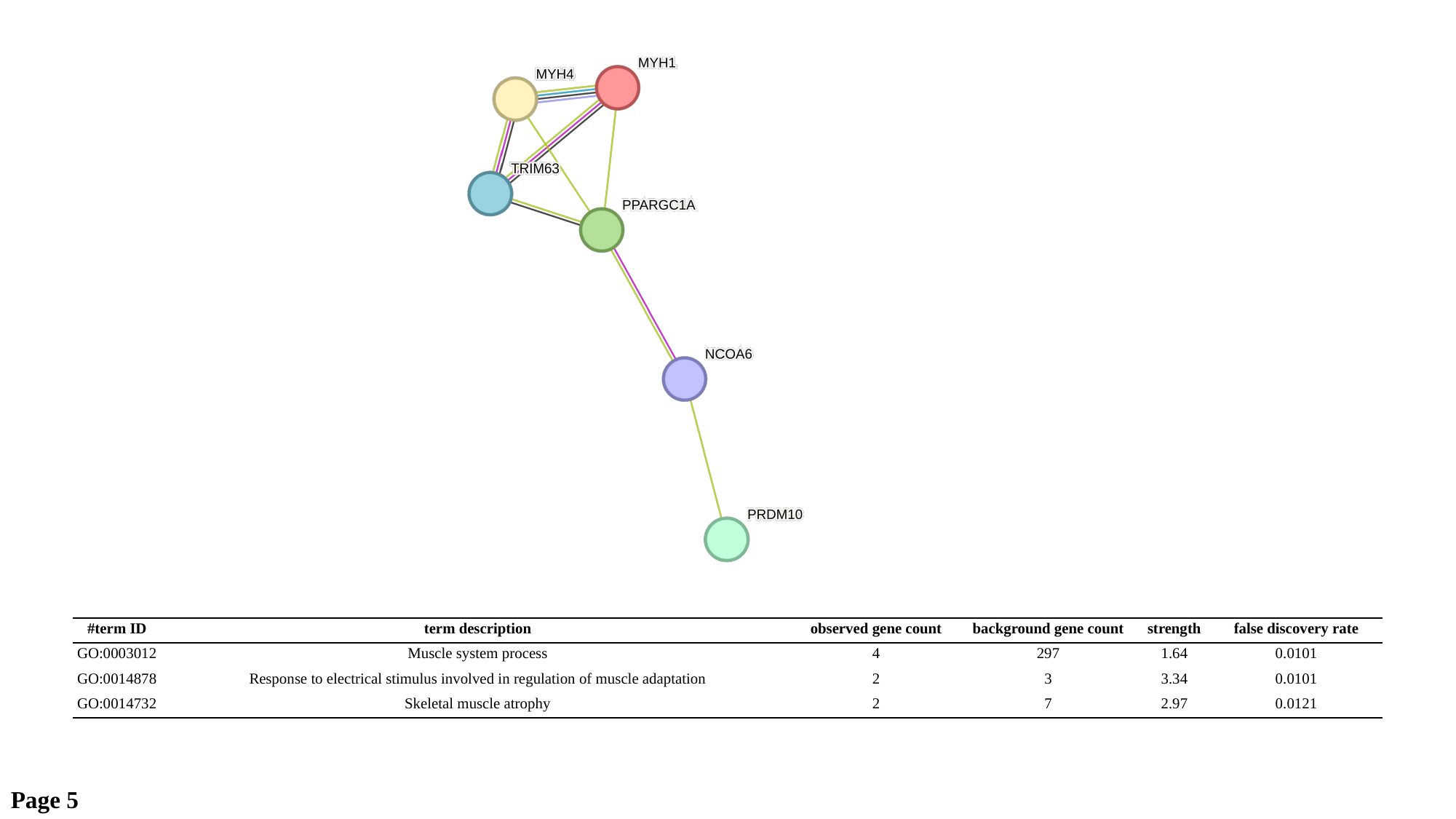

| #term ID | term description | observed gene count | background gene count | strength | false discovery rate |
| --- | --- | --- | --- | --- | --- |
| GO:0003012 | Muscle system process | 4 | 297 | 1.64 | 0.0101 |
| GO:0014878 | Response to electrical stimulus involved in regulation of muscle adaptation | 2 | 3 | 3.34 | 0.0101 |
| GO:0014732 | Skeletal muscle atrophy | 2 | 7 | 2.97 | 0.0121 |
Page 5

## Slide 6
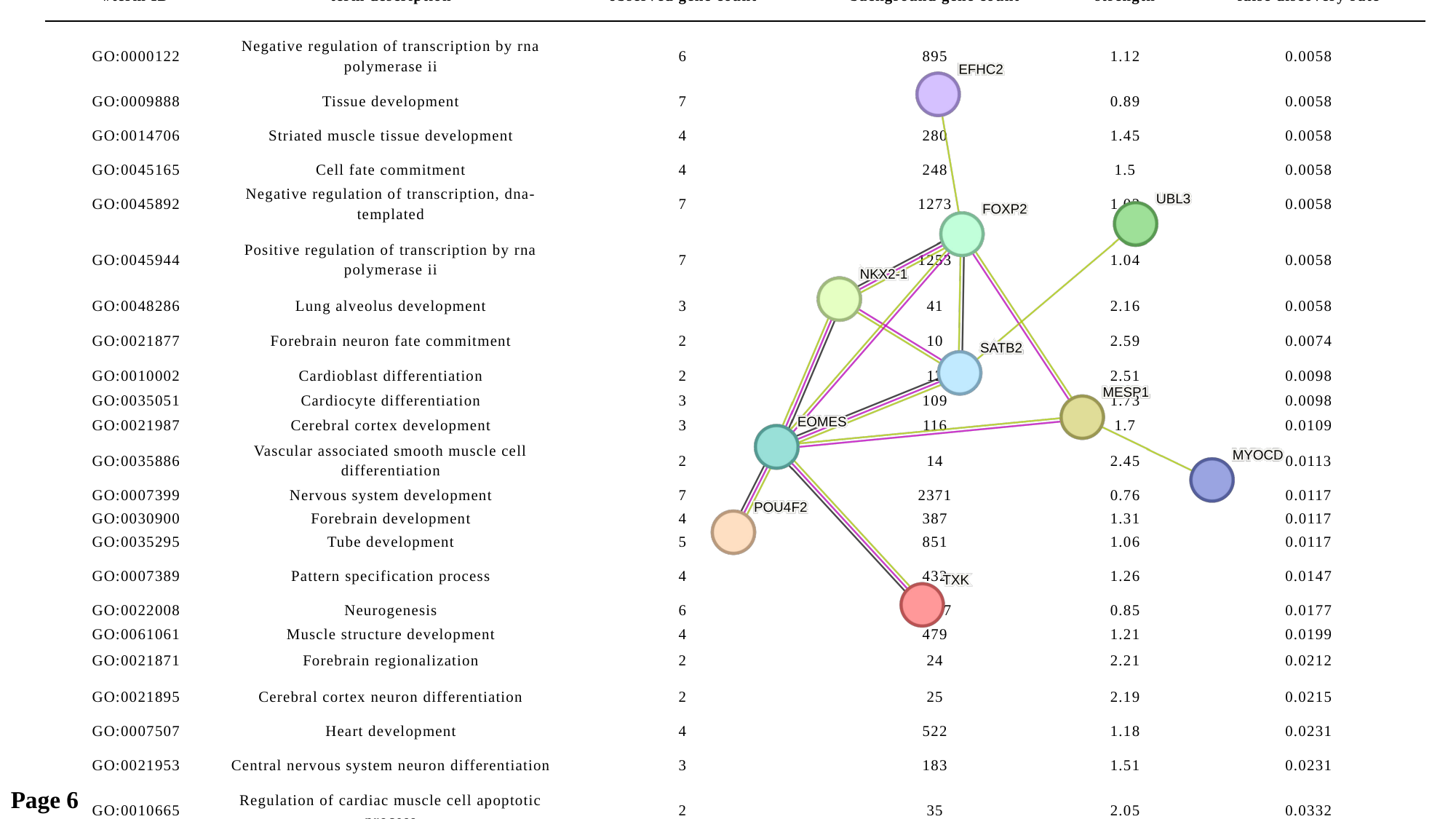

| #term ID | term description | observed gene count | background gene count | strength | false discovery rate |
| --- | --- | --- | --- | --- | --- |
| GO:0000122 | Negative regulation of transcription by rna polymerase ii | 6 | 895 | 1.12 | 0.0058 |
| GO:0009888 | Tissue development | 7 | 1760 | 0.89 | 0.0058 |
| GO:0014706 | Striated muscle tissue development | 4 | 280 | 1.45 | 0.0058 |
| GO:0045165 | Cell fate commitment | 4 | 248 | 1.5 | 0.0058 |
| GO:0045892 | Negative regulation of transcription, dna-templated | 7 | 1273 | 1.03 | 0.0058 |
| GO:0045944 | Positive regulation of transcription by rna polymerase ii | 7 | 1253 | 1.04 | 0.0058 |
| GO:0048286 | Lung alveolus development | 3 | 41 | 2.16 | 0.0058 |
| GO:0021877 | Forebrain neuron fate commitment | 2 | 10 | 2.59 | 0.0074 |
| GO:0010002 | Cardioblast differentiation | 2 | 12 | 2.51 | 0.0098 |
| GO:0035051 | Cardiocyte differentiation | 3 | 109 | 1.73 | 0.0098 |
| GO:0021987 | Cerebral cortex development | 3 | 116 | 1.7 | 0.0109 |
| GO:0035886 | Vascular associated smooth muscle cell differentiation | 2 | 14 | 2.45 | 0.0113 |
| GO:0007399 | Nervous system development | 7 | 2371 | 0.76 | 0.0117 |
| GO:0030900 | Forebrain development | 4 | 387 | 1.31 | 0.0117 |
| GO:0035295 | Tube development | 5 | 851 | 1.06 | 0.0117 |
| GO:0007389 | Pattern specification process | 4 | 432 | 1.26 | 0.0147 |
| GO:0022008 | Neurogenesis | 6 | 1657 | 0.85 | 0.0177 |
| GO:0061061 | Muscle structure development | 4 | 479 | 1.21 | 0.0199 |
| GO:0021871 | Forebrain regionalization | 2 | 24 | 2.21 | 0.0212 |
| GO:0021895 | Cerebral cortex neuron differentiation | 2 | 25 | 2.19 | 0.0215 |
| GO:0007507 | Heart development | 4 | 522 | 1.18 | 0.0231 |
| GO:0021953 | Central nervous system neuron differentiation | 3 | 183 | 1.51 | 0.0231 |
| GO:0010665 | Regulation of cardiac muscle cell apoptotic process | 2 | 35 | 2.05 | 0.0332 |
| GO:0048731 | System development | 8 | 4426 | 0.55 | 0.0411 |
| GO:2000026 | Regulation of multicellular organismal development | 6 | 2096 | 0.75 | 0.0437 |
| GO:0035239 | Tube morphogenesis | 4 | 656 | 1.08 | 0.0452 |
| GO:0048513 | Animal organ development | 7 | 3197 | 0.63 | 0.0461 |
| GO:0051239 | Regulation of multicellular organismal process | 7 | 3227 | 0.63 | 0.0482 |
| GO:0009653 | Anatomical structure morphogenesis | 6 | 2165 | 0.73 | 0.0487 |
Page 6

## Slide 7
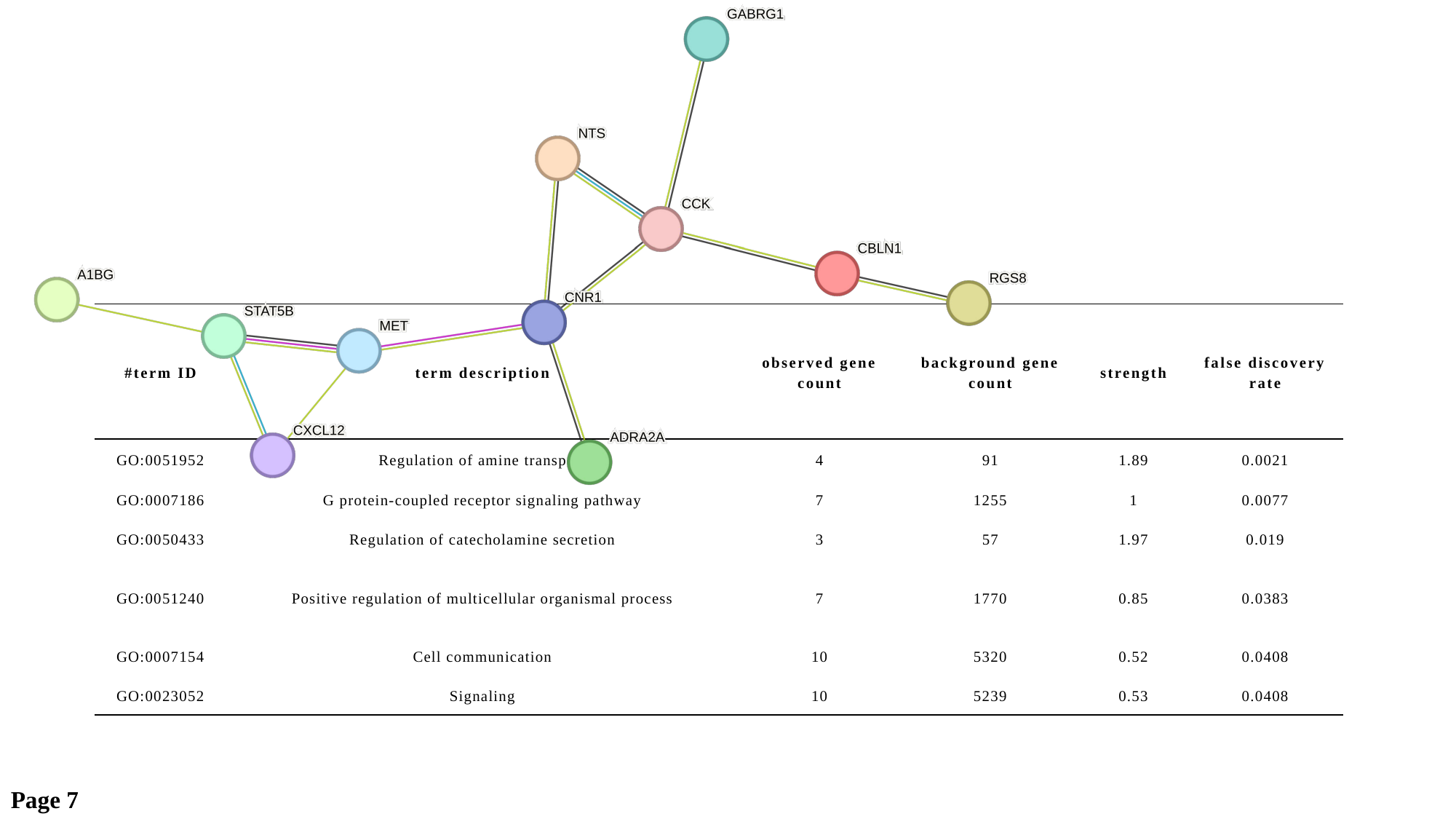

| #term ID | term description | observed gene count | background gene count | strength | false discovery rate |
| --- | --- | --- | --- | --- | --- |
| GO:0051952 | Regulation of amine transport | 4 | 91 | 1.89 | 0.0021 |
| GO:0007186 | G protein-coupled receptor signaling pathway | 7 | 1255 | 1 | 0.0077 |
| GO:0050433 | Regulation of catecholamine secretion | 3 | 57 | 1.97 | 0.019 |
| GO:0051240 | Positive regulation of multicellular organismal process | 7 | 1770 | 0.85 | 0.0383 |
| GO:0007154 | Cell communication | 10 | 5320 | 0.52 | 0.0408 |
| GO:0023052 | Signaling | 10 | 5239 | 0.53 | 0.0408 |
Page 7

## Slide 8
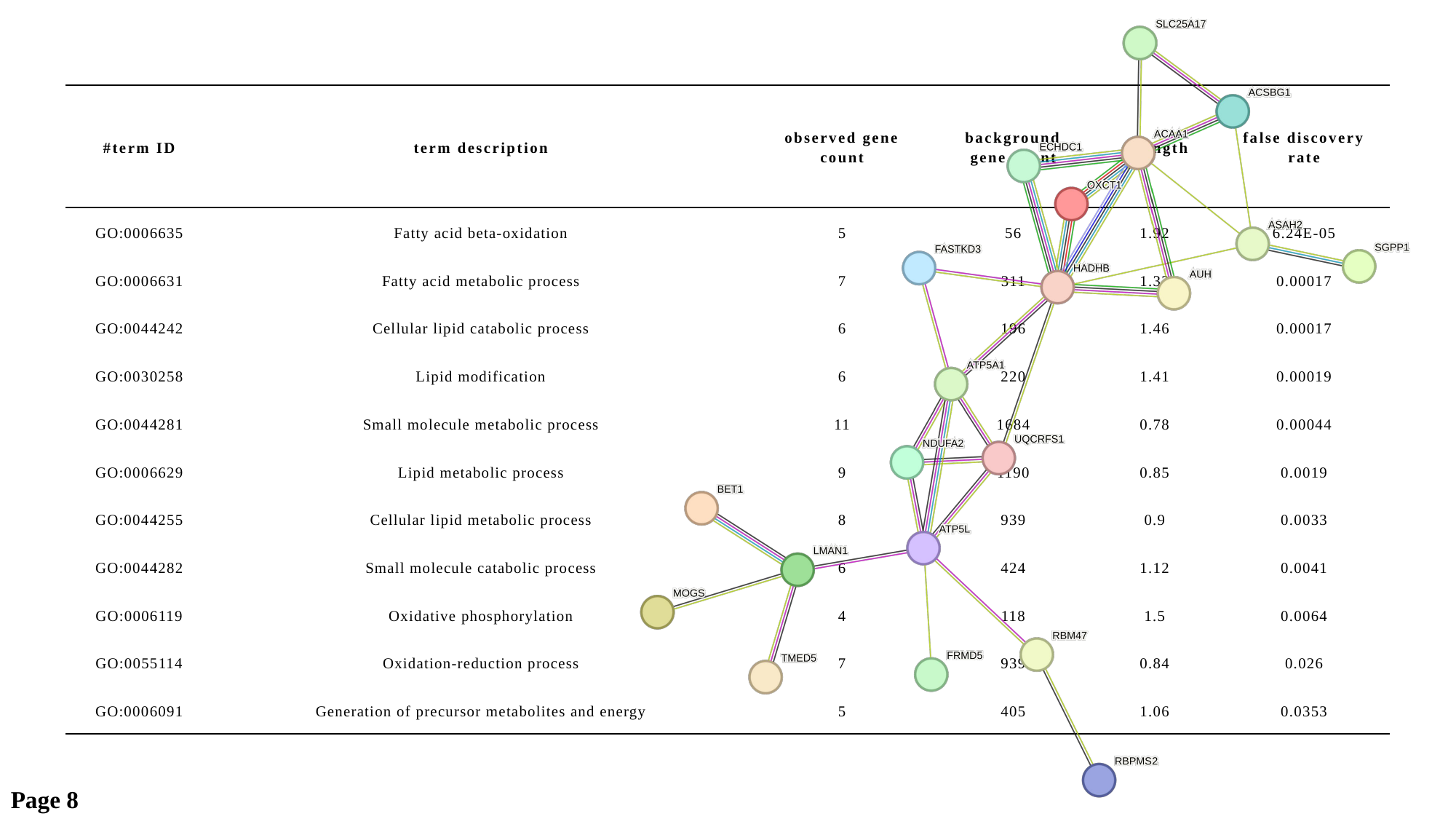

| #term ID | term description | observed gene count | background gene count | strength | false discovery rate |
| --- | --- | --- | --- | --- | --- |
| GO:0006635 | Fatty acid beta-oxidation | 5 | 56 | 1.92 | 6.24E-05 |
| GO:0006631 | Fatty acid metabolic process | 7 | 311 | 1.32 | 0.00017 |
| GO:0044242 | Cellular lipid catabolic process | 6 | 196 | 1.46 | 0.00017 |
| GO:0030258 | Lipid modification | 6 | 220 | 1.41 | 0.00019 |
| GO:0044281 | Small molecule metabolic process | 11 | 1684 | 0.78 | 0.00044 |
| GO:0006629 | Lipid metabolic process | 9 | 1190 | 0.85 | 0.0019 |
| GO:0044255 | Cellular lipid metabolic process | 8 | 939 | 0.9 | 0.0033 |
| GO:0044282 | Small molecule catabolic process | 6 | 424 | 1.12 | 0.0041 |
| GO:0006119 | Oxidative phosphorylation | 4 | 118 | 1.5 | 0.0064 |
| GO:0055114 | Oxidation-reduction process | 7 | 939 | 0.84 | 0.026 |
| GO:0006091 | Generation of precursor metabolites and energy | 5 | 405 | 1.06 | 0.0353 |
Page 8

## Slide 9
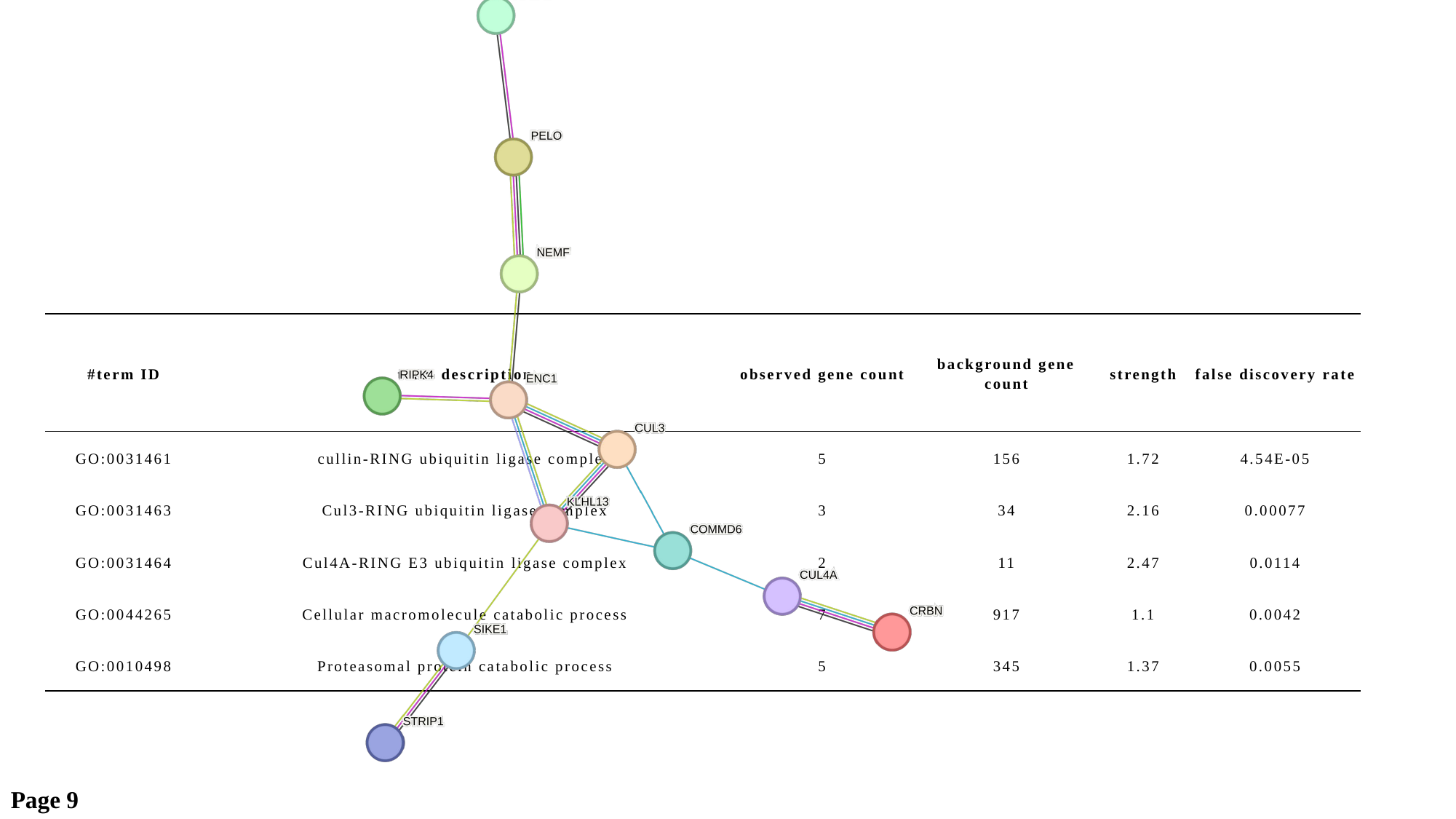

| #term ID | term description | observed gene count | background gene count | strength | false discovery rate |
| --- | --- | --- | --- | --- | --- |
| GO:0031461 | cullin-RING ubiquitin ligase complex | 5 | 156 | 1.72 | 4.54E-05 |
| GO:0031463 | Cul3-RING ubiquitin ligase complex | 3 | 34 | 2.16 | 0.00077 |
| GO:0031464 | Cul4A-RING E3 ubiquitin ligase complex | 2 | 11 | 2.47 | 0.0114 |
| GO:0044265 | Cellular macromolecule catabolic process | 7 | 917 | 1.1 | 0.0042 |
| GO:0010498 | Proteasomal protein catabolic process | 5 | 345 | 1.37 | 0.0055 |
Page 9

## Slide 10
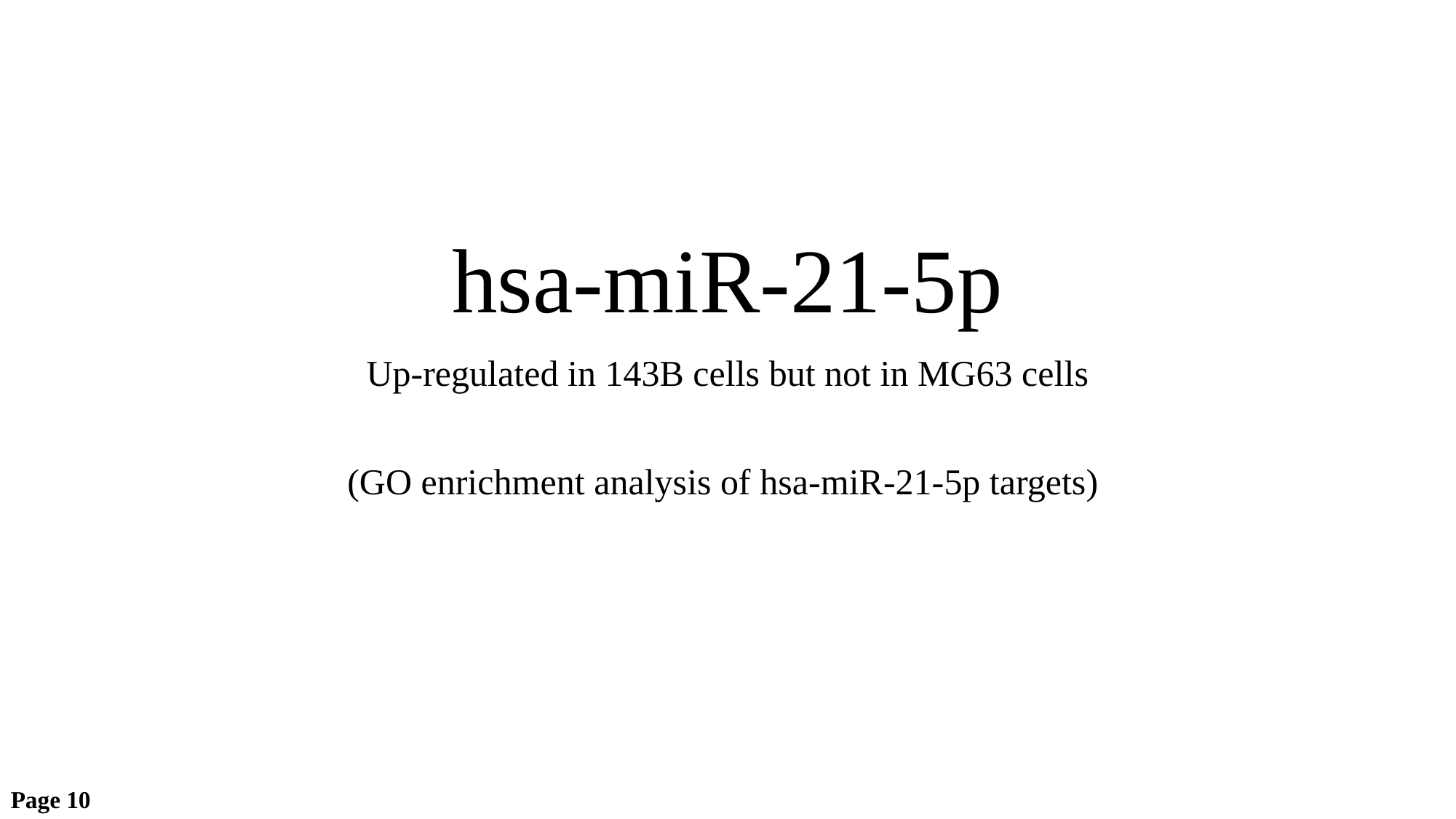

# hsa-miR-21-5p
Up-regulated in 143B cells but not in MG63 cells
(GO enrichment analysis of hsa-miR-21-5p targets)
Page 10

## Slide 11
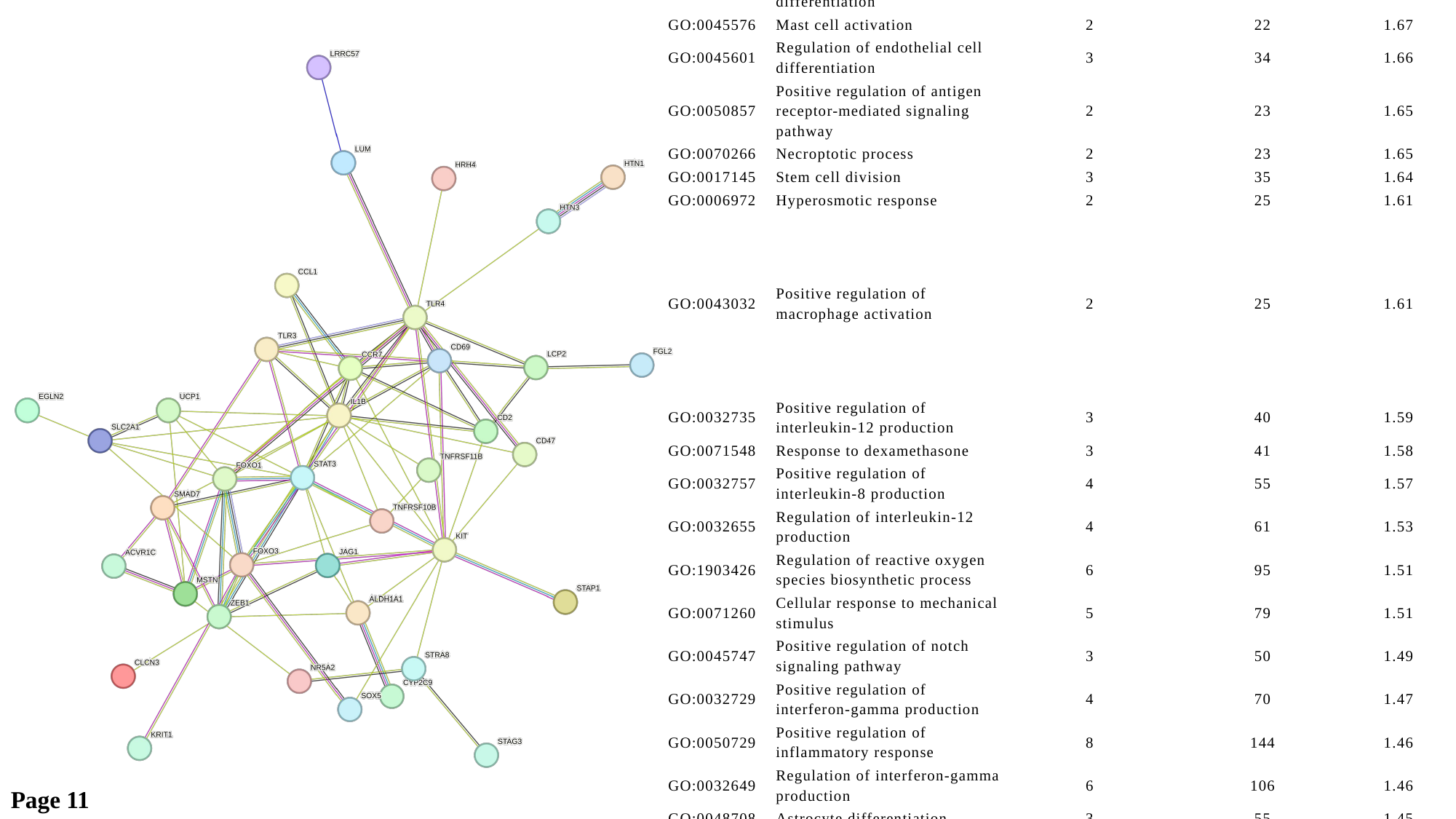

| #term ID | term description | observed gene count | background gene count | strength |
| --- | --- | --- | --- | --- |
| GO:0070487 | Monocyte aggregation | 2 | 4 | 2.41 |
| GO:1903972 | Regulation of cellular response to macrophage colony-stimulating factor stimulus | 2 | 5 | 2.31 |
| GO:0034128 | Negative regulation of myd88-independent toll-like receptor signaling pathway | 2 | 7 | 2.17 |
| GO:0035234 | Ectopic germ cell programmed cell death | 2 | 7 | 2.17 |
| GO:0045602 | Negative regulation of endothelial cell differentiation | 2 | 9 | 2.06 |
| GO:0098722 | Asymmetric stem cell division | 2 | 10 | 2.01 |
| GO:0002604 | Regulation of dendritic cell antigen processing and presentation | 2 | 11 | 1.97 |
| GO:0070417 | Cellular response to cold | 2 | 11 | 1.97 |
| GO:0002730 | Regulation of dendritic cell cytokine production | 2 | 12 | 1.93 |
| GO:0007252 | I-kappaB phosphorylation | 2 | 13 | 1.9 |
| GO:0014842 | Regulation of skeletal muscle satellite cell proliferation | 2 | 13 | 1.9 |
| GO:0031274 | Positive regulation of pseudopodium assembly | 2 | 13 | 1.9 |
| GO:0032611 | interleukin-1 beta production | 2 | 15 | 1.84 |
| GO:0097150 | Neuronal stem cell population maintenance | 3 | 23 | 1.83 |
| GO:0002922 | Positive regulation of humoral immune response | 2 | 17 | 1.78 |
| GO:0007250 | Activation of nf-kappab-inducing kinase activity | 2 | 17 | 1.78 |
| GO:1903209 | Positive regulation of oxidative stress-induced cell death | 2 | 17 | 1.78 |
| GO:0032332 | Positive regulation of chondrocyte differentiation | 2 | 20 | 1.71 |
| GO:0071731 | Response to nitric oxide | 2 | 20 | 1.71 |
| GO:0001659 | Temperature homeostasis | 3 | 31 | 1.7 |
| GO:1900017 | Positive regulation of cytokine production involved in inflammatory response | 2 | 21 | 1.69 |
| GO:2000737 | Negative regulation of stem cell differentiation | 2 | 21 | 1.69 |
| GO:0045576 | Mast cell activation | 2 | 22 | 1.67 |
| GO:0045601 | Regulation of endothelial cell differentiation | 3 | 34 | 1.66 |
| GO:0050857 | Positive regulation of antigen receptor-mediated signaling pathway | 2 | 23 | 1.65 |
| GO:0070266 | Necroptotic process | 2 | 23 | 1.65 |
| GO:0017145 | Stem cell division | 3 | 35 | 1.64 |
| GO:0006972 | Hyperosmotic response | 2 | 25 | 1.61 |
| GO:0043032 | Positive regulation of macrophage activation | 2 | 25 | 1.61 |
| GO:0032735 | Positive regulation of interleukin-12 production | 3 | 40 | 1.59 |
| GO:0071548 | Response to dexamethasone | 3 | 41 | 1.58 |
| GO:0032757 | Positive regulation of interleukin-8 production | 4 | 55 | 1.57 |
| GO:0032655 | Regulation of interleukin-12 production | 4 | 61 | 1.53 |
| GO:1903426 | Regulation of reactive oxygen species biosynthetic process | 6 | 95 | 1.51 |
| GO:0071260 | Cellular response to mechanical stimulus | 5 | 79 | 1.51 |
| GO:0045747 | Positive regulation of notch signaling pathway | 3 | 50 | 1.49 |
| GO:0032729 | Positive regulation of interferon-gamma production | 4 | 70 | 1.47 |
| GO:0050729 | Positive regulation of inflammatory response | 8 | 144 | 1.46 |
| GO:0032649 | Regulation of interferon-gamma production | 6 | 106 | 1.46 |
| GO:0048708 | Astrocyte differentiation | 3 | 55 | 1.45 |
| GO:1903428 | Positive regulation of reactive oxygen species biosynthetic process | 3 | 56 | 1.44 |
| GO:0071385 | Cellular response to glucocorticoid stimulus | 3 | 59 | 1.42 |
| GO:0035690 | Cellular response to drug | 3 | 60 | 1.41 |
| GO:1901224 | Positive regulation of nik/nf-kappab signaling | 4 | 82 | 1.4 |
| GO:0009620 | Response to fungus | 3 | 61 | 1.4 |
| GO:0032722 | Positive regulation of chemokine production | 3 | 61 | 1.4 |
| GO:0045428 | Regulation of nitric oxide biosynthetic process | 3 | 61 | 1.4 |
| GO:0050766 | Positive regulation of phagocytosis | 3 | 66 | 1.37 |
| GO:0002718 | Regulation of cytokine production involved in immune response | 4 | 89 | 1.36 |
| GO:0070542 | Response to fatty acid | 4 | 91 | 1.35 |
| GO:0032755 | Positive regulation of interleukin-6 production | 4 | 94 | 1.34 |
| GO:0097529 | Myeloid leukocyte migration | 5 | 123 | 1.32 |
| GO:0097191 | Extrinsic apoptotic signaling pathway | 4 | 100 | 1.31 |
| GO:1902106 | Negative regulation of leukocyte differentiation | 4 | 104 | 1.3 |
| GO:2000377 | Regulation of reactive oxygen species metabolic process | 7 | 188 | 1.28 |
| GO:0019827 | Stem cell population maintenance | 5 | 135 | 1.28 |
| GO:0032675 | Regulation of interleukin-6 production | 5 | 142 | 1.26 |
| GO:0045580 | Regulation of t cell differentiation | 5 | 147 | 1.24 |
| GO:0032760 | Positive regulation of tumor necrosis factor production | 3 | 88 | 1.24 |
| GO:0010717 | Regulation of epithelial to mesenchymal transition | 3 | 90 | 1.23 |
| GO:0031640 | Killing of cells of other organism | 3 | 91 | 1.23 |
| GO:0031214 | Biomineral tissue development | 3 | 92 | 1.23 |
| GO:0002688 | Regulation of leukocyte chemotaxis | 4 | 124 | 1.22 |
| GO:0001816 | Cytokine production | 5 | 158 | 1.21 |
| GO:1901655 | Cellular response to ketone | 3 | 95 | 1.21 |
| GO:0002690 | Positive regulation of leukocyte chemotaxis | 3 | 98 | 1.2 |
| GO:0045639 | Positive regulation of myeloid cell differentiation | 3 | 99 | 1.19 |
| GO:0046330 | Positive regulation of jnk cascade | 4 | 137 | 1.18 |
| GO:0120034 | Positive regulation of plasma membrane bounded cell projection assembly | 3 | 104 | 1.17 |
| GO:0009612 | Response to mechanical stimulus | 6 | 212 | 1.16 |
| GO:0030595 | Leukocyte chemotaxis | 4 | 142 | 1.16 |
| GO:0030336 | Negative regulation of cell migration | 7 | 262 | 1.14 |
| GO:0071383 | Cellular response to steroid hormone stimulus | 5 | 188 | 1.14 |
| GO:0002460 | Adaptive immune response based on somatic recombination of immune receptors built from immunoglobulin superfamily domains | 4 | 148 | 1.14 |
| GO:0032680 | Regulation of tumor necrosis factor production | 4 | 152 | 1.13 |
| GO:0043123 | Positive regulation of i-kappab kinase/nf-kappab signaling | 5 | 194 | 1.12 |
| GO:0051092 | Positive regulation of nf-kappab transcription factor activity | 4 | 158 | 1.12 |
| GO:0071346 | Cellular response to interferon-gamma | 4 | 161 | 1.11 |
| GO:1902105 | Regulation of leukocyte differentiation | 7 | 285 | 1.1 |
| GO:0002703 | Regulation of leukocyte mediated immunity | 5 | 209 | 1.09 |
| GO:0071496 | Cellular response to external stimulus | 7 | 309 | 1.07 |
| GO:0050900 | Leukocyte migration | 7 | 316 | 1.06 |
| GO:0120032 | Regulation of plasma membrane bounded cell projection assembly | 4 | 181 | 1.06 |
| GO:0032872 | Regulation of stress-activated mapk cascade | 5 | 228 | 1.05 |
| GO:0090257 | Regulation of muscle system process | 5 | 231 | 1.05 |
| GO:0050863 | Regulation of t cell activation | 7 | 329 | 1.04 |
| GO:1904018 | Positive regulation of vasculature development | 4 | 189 | 1.04 |
| GO:0042110 | T cell activation | 5 | 243 | 1.03 |
| GO:1903706 | Regulation of hemopoiesis | 10 | 493 | 1.02 |
| GO:1901654 | Response to ketone | 4 | 199 | 1.01 |
| GO:1903708 | Positive regulation of hemopoiesis | 4 | 199 | 1.01 |
| GO:0045637 | Regulation of myeloid cell differentiation | 5 | 260 | 1 |
| GO:0071396 | Cellular response to lipid | 10 | 528 | 0.99 |
| GO:0051091 | Positive regulation of dna-binding transcription factor activity | 5 | 271 | 0.98 |
| GO:0051249 | Regulation of lymphocyte activation | 8 | 445 | 0.97 |
| GO:0002696 | Positive regulation of leukocyte activation | 6 | 332 | 0.97 |
| GO:0032103 | Positive regulation of response to external stimulus | 9 | 511 | 0.96 |
| GO:0042493 | Response to drug | 5 | 281 | 0.96 |
| GO:0006954 | Inflammatory response | 9 | 515 | 0.95 |
| GO:0001819 | Positive regulation of cytokine production | 8 | 461 | 0.95 |
| GO:0097190 | Apoptotic signaling pathway | 5 | 286 | 0.95 |
| GO:0050768 | Negative regulation of neurogenesis | 5 | 287 | 0.95 |
| GO:0032868 | Response to insulin | 4 | 231 | 0.95 |
| GO:0097305 | Response to alcohol | 4 | 233 | 0.95 |
| GO:0002694 | Regulation of leukocyte activation | 9 | 530 | 0.94 |
| GO:0071407 | Cellular response to organic cyclic compound | 9 | 537 | 0.94 |
| GO:0002697 | Regulation of immune effector process | 7 | 418 | 0.94 |
| GO:0045596 | Negative regulation of cell differentiation | 12 | 728 | 0.93 |
| GO:0045732 | Positive regulation of protein catabolic process | 4 | 240 | 0.93 |
| GO:1903037 | Regulation of leukocyte cell-cell adhesion | 5 | 315 | 0.91 |
| GO:0051093 | Negative regulation of developmental process | 15 | 983 | 0.9 |
| GO:0042326 | Negative regulation of phosphorylation | 7 | 449 | 0.9 |
| GO:0046649 | Lymphocyte activation | 6 | 390 | 0.9 |
| GO:1901652 | Response to peptide | 7 | 476 | 0.88 |
| GO:1901342 | Regulation of vasculature development | 5 | 336 | 0.88 |
| GO:0045860 | Positive regulation of protein kinase activity | 8 | 550 | 0.87 |
| GO:0009725 | Response to hormone | 12 | 849 | 0.86 |
| GO:0033993 | Response to lipid | 12 | 858 | 0.86 |
| GO:0032870 | Cellular response to hormone stimulus | 8 | 569 | 0.86 |
| GO:0022407 | Regulation of cell-cell adhesion | 6 | 424 | 0.86 |
| GO:0071345 | Cellular response to cytokine stimulus | 14 | 1013 | 0.85 |
| GO:0030334 | Regulation of cell migration | 12 | 865 | 0.85 |
| GO:0051090 | Regulation of dna-binding transcription factor activity | 6 | 437 | 0.85 |
| GO:0001817 | Regulation of cytokine production | 10 | 742 | 0.84 |
| GO:0031347 | Regulation of defense response | 9 | 674 | 0.84 |
| GO:0031667 | Response to nutrient levels | 6 | 449 | 0.84 |
| GO:0002683 | Negative regulation of immune system process | 6 | 450 | 0.84 |
| GO:0032147 | Activation of protein kinase activity | 5 | 376 | 0.84 |
| GO:0051241 | Negative regulation of multicellular organismal process | 16 | 1231 | 0.83 |
| GO:0019221 | Cytokine-mediated signaling pathway | 9 | 678 | 0.83 |
| GO:0045862 | Positive regulation of proteolysis | 5 | 377 | 0.83 |
| GO:0043434 | Response to peptide hormone | 5 | 394 | 0.82 |
| GO:0002684 | Positive regulation of immune system process | 12 | 949 | 0.81 |
| GO:0007167 | Enzyme linked receptor protein signaling pathway | 9 | 720 | 0.81 |
| GO:0009617 | Response to bacterium | 8 | 634 | 0.81 |
| GO:0009719 | Response to endogenous stimulus | 17 | 1447 | 0.78 |
| GO:0007169 | Transmembrane receptor protein tyrosine kinase signaling pathway | 6 | 518 | 0.78 |
| GO:0030335 | Positive regulation of cell migration | 6 | 522 | 0.77 |
| GO:0070848 | Response to growth factor | 6 | 524 | 0.77 |
| GO:0002682 | Regulation of immune system process | 17 | 1514 | 0.76 |
| GO:1901698 | Response to nitrogen compound | 12 | 1070 | 0.76 |
| GO:0050776 | Regulation of immune response | 10 | 896 | 0.76 |
| GO:0043410 | Positive regulation of mapk cascade | 6 | 543 | 0.76 |
| GO:0071495 | Cellular response to endogenous stimulus | 13 | 1181 | 0.75 |
| GO:0014070 | Response to organic cyclic compound | 10 | 911 | 0.75 |
| GO:0043408 | Regulation of mapk cascade | 8 | 725 | 0.75 |
| GO:1901699 | Cellular response to nitrogen compound | 7 | 645 | 0.75 |
| GO:0001934 | Positive regulation of protein phosphorylation | 11 | 1019 | 0.74 |
| GO:0045944 | Positive regulation of transcription by rna polymerase ii | 13 | 1253 | 0.73 |
| GO:1901701 | Cellular response to oxygen-containing compound | 11 | 1055 | 0.73 |
| GO:0010243 | Response to organonitrogen compound | 10 | 987 | 0.72 |
| GO:0002274 | Myeloid leukocyte activation | 6 | 585 | 0.72 |
| GO:0071417 | Cellular response to organonitrogen compound | 6 | 590 | 0.72 |
| GO:0044057 | Regulation of system process | 6 | 592 | 0.72 |
| GO:0032101 | Regulation of response to external stimulus | 10 | 1013 | 0.71 |
| GO:0019725 | Cellular homeostasis | 9 | 895 | 0.71 |
| GO:0008285 | Negative regulation of cell population proliferation | 7 | 696 | 0.71 |
| GO:0051338 | Regulation of transferase activity | 10 | 1036 | 0.7 |
| GO:0006915 | Apoptotic process | 9 | 918 | 0.7 |
| GO:0043549 | Regulation of kinase activity | 9 | 918 | 0.7 |
| GO:0045321 | Leukocyte activation | 9 | 929 | 0.7 |
| GO:1901700 | Response to oxygen-containing compound | 15 | 1567 | 0.69 |
| GO:0001932 | Regulation of protein phosphorylation | 14 | 1459 | 0.69 |
| GO:0031401 | Positive regulation of protein modification process | 12 | 1252 | 0.69 |
| GO:0009628 | Response to abiotic stimulus | 11 | 1147 | 0.69 |
| GO:0012501 | Programmed cell death | 10 | 1054 | 0.69 |
| GO:0071310 | Cellular response to organic substance | 22 | 2369 | 0.68 |
| GO:0001775 | Cell activation | 10 | 1075 | 0.68 |
| GO:0007166 | Cell surface receptor signaling pathway | 21 | 2325 | 0.67 |
| GO:0051345 | Positive regulation of hydrolase activity | 7 | 772 | 0.67 |
| GO:0042592 | Homeostatic process | 15 | 1676 | 0.66 |
| GO:0045893 | Positive regulation of transcription, dna-templated | 14 | 1587 | 0.66 |
| GO:0016477 | Cell migration | 8 | 896 | 0.66 |
| GO:0051247 | Positive regulation of protein metabolic process | 15 | 1715 | 0.65 |
| GO:0010648 | Negative regulation of cell communication | 12 | 1382 | 0.65 |
| GO:0023057 | Negative regulation of signaling | 12 | 1385 | 0.65 |
| GO:0009968 | Negative regulation of signal transduction | 11 | 1271 | 0.65 |
| GO:0070887 | Cellular response to chemical stimulus | 25 | 2919 | 0.64 |
| GO:0051240 | Positive regulation of multicellular organismal process | 15 | 1770 | 0.64 |
| GO:0042127 | Regulation of cell population proliferation | 14 | 1642 | 0.64 |
| GO:0006952 | Defense response | 11 | 1296 | 0.64 |
| GO:0050767 | Regulation of neurogenesis | 7 | 828 | 0.64 |
| GO:0010033 | Response to organic substance | 25 | 3011 | 0.63 |
| GO:0060284 | Regulation of cell development | 8 | 956 | 0.63 |
| GO:0009887 | Animal organ morphogenesis | 8 | 967 | 0.63 |
| GO:0010628 | Positive regulation of gene expression | 19 | 2337 | 0.62 |
| GO:2000026 | Regulation of multicellular organismal development | 17 | 2096 | 0.62 |
| GO:0045595 | Regulation of cell differentiation | 15 | 1874 | 0.62 |
| GO:0006955 | Immune response | 13 | 1588 | 0.62 |
| GO:0043085 | Positive regulation of catalytic activity | 12 | 1489 | 0.62 |
| GO:0048878 | Chemical homeostasis | 9 | 1124 | 0.62 |
| GO:0045597 | Positive regulation of cell differentiation | 8 | 993 | 0.62 |
| GO:0010557 | Positive regulation of macromolecule biosynthetic process | 15 | 1906 | 0.61 |
| GO:0032270 | Positive regulation of cellular protein metabolic process | 13 | 1635 | 0.61 |
| GO:0080134 | Regulation of response to stress | 11 | 1437 | 0.6 |
| GO:0031328 | Positive regulation of cellular biosynthetic process | 15 | 2005 | 0.59 |
| GO:0051239 | Regulation of multicellular organismal process | 24 | 3227 | 0.58 |
| GO:0009605 | Response to external stimulus | 17 | 2310 | 0.58 |
| GO:0006357 | Regulation of transcription by rna polymerase ii | 16 | 2172 | 0.58 |
| GO:0048585 | Negative regulation of response to stimulus | 12 | 1636 | 0.58 |
| GO:0051707 | Response to other organism | 9 | 1256 | 0.57 |
| GO:0010941 | Regulation of cell death | 12 | 1696 | 0.56 |
| GO:0051173 | Positive regulation of nitrogen compound metabolic process | 22 | 3239 | 0.54 |
| GO:0050793 | Regulation of developmental process | 18 | 2648 | 0.54 |
| GO:0009967 | Positive regulation of signal transduction | 11 | 1654 | 0.53 |
| GO:0010604 | Positive regulation of macromolecule metabolic process | 23 | 3600 | 0.52 |
| GO:0043067 | Regulation of programmed cell death | 10 | 1569 | 0.52 |
| GO:0051094 | Positive regulation of developmental process | 9 | 1389 | 0.52 |
| GO:0022414 | Reproductive process | 9 | 1400 | 0.52 |
| GO:0042221 | Response to chemical | 27 | 4333 | 0.51 |
| GO:0050790 | Regulation of catalytic activity | 15 | 2386 | 0.51 |
| GO:0009893 | Positive regulation of metabolic process | 24 | 3893 | 0.5 |
| GO:0031325 | Positive regulation of cellular metabolic process | 21 | 3413 | 0.5 |
| GO:0048584 | Positive regulation of response to stimulus | 14 | 2257 | 0.5 |
| GO:0051246 | Regulation of protein metabolic process | 17 | 2828 | 0.49 |
| GO:0032268 | Regulation of cellular protein metabolic process | 16 | 2693 | 0.49 |
| GO:0002376 | Immune system process | 15 | 2481 | 0.49 |
| GO:0035556 | Intracellular signal transduction | 10 | 1712 | 0.48 |
| GO:0006950 | Response to stress | 20 | 3485 | 0.47 |
| GO:0065008 | Regulation of biological quality | 22 | 4042 | 0.45 |
| GO:0009966 | Regulation of signal transduction | 17 | 3107 | 0.45 |
| GO:0032879 | Regulation of localization | 15 | 2740 | 0.45 |
| GO:0010646 | Regulation of cell communication | 19 | 3514 | 0.44 |
| GO:0023051 | Regulation of signaling | 19 | 3553 | 0.44 |
| GO:0048731 | System development | 22 | 4426 | 0.41 |
| GO:0006355 | Regulation of transcription, dna-templated | 17 | 3388 | 0.41 |
| GO:0048513 | Animal organ development | 16 | 3197 | 0.41 |
| GO:0007165 | Signal transduction | 24 | 4876 | 0.4 |
| GO:0051716 | Cellular response to stimulus | 30 | 6489 | 0.38 |
| GO:0007154 | Cell communication | 25 | 5320 | 0.38 |
| GO:0048583 | Regulation of response to stimulus | 19 | 4114 | 0.38 |
| GO:0031326 | Regulation of cellular biosynthetic process | 19 | 4125 | 0.38 |
| GO:2000112 | Regulation of cellular macromolecule biosynthetic process | 18 | 3878 | 0.38 |
| GO:0010468 | Regulation of gene expression | 22 | 4813 | 0.37 |
| GO:0065009 | Regulation of molecular function | 22 | 4913 | 0.36 |
| GO:0050896 | Response to stimulus | 34 | 8046 | 0.34 |
| GO:0051171 | Regulation of nitrogen compound metabolic process | 25 | 5836 | 0.34 |
| GO:0048856 | Anatomical structure development | 23 | 5402 | 0.34 |
| GO:0032502 | Developmental process | 24 | 5841 | 0.33 |
| GO:0048522 | Positive regulation of cellular process | 23 | 5579 | 0.33 |
| GO:0060255 | Regulation of macromolecule metabolic process | 26 | 6407 | 0.32 |
| GO:0048518 | Positive regulation of biological process | 25 | 6112 | 0.32 |
| GO:0031323 | Regulation of cellular metabolic process | 25 | 6239 | 0.31 |
| GO:0080090 | Regulation of primary metabolic process | 24 | 6032 | 0.31 |
| GO:0019222 | Regulation of metabolic process | 27 | 6948 | 0.3 |
| GO:0048519 | Negative regulation of biological process | 21 | 5389 | 0.3 |
| GO:0048523 | Negative regulation of cellular process | 19 | 4874 | 0.3 |
| GO:0032501 | Multicellular organismal process | 26 | 6933 | 0.29 |
| GO:0065007 | Biological regulation | 33 | 12171 | 0.14 |
| GO:0009987 | Cellular process | 37 | 15024 | 0.1 |
Page 11

## Slide 12
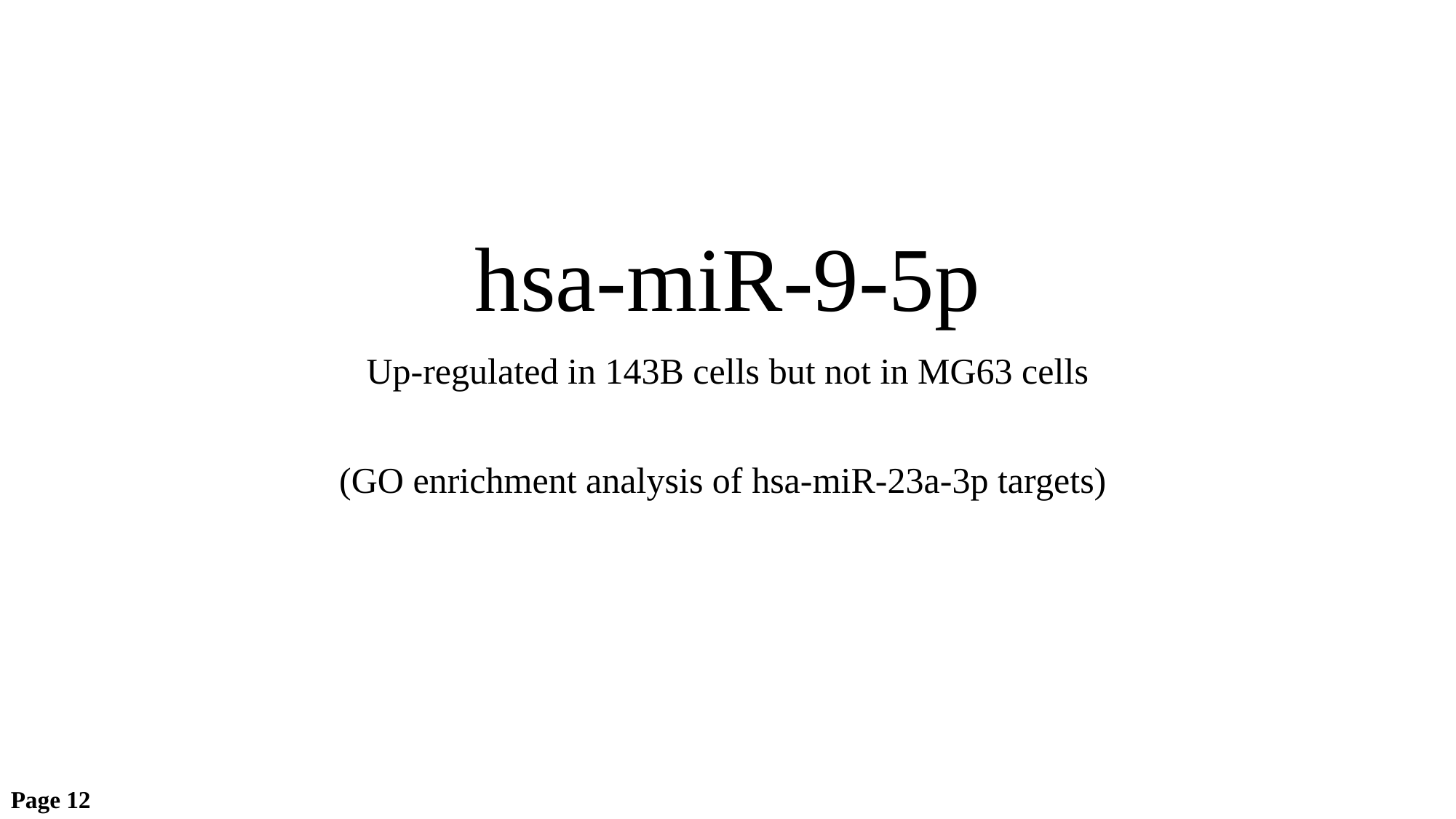

# hsa-miR-9-5p
Up-regulated in 143B cells but not in MG63 cells
(GO enrichment analysis of hsa-miR-23a-3p targets)
Page 12

## Slide 13
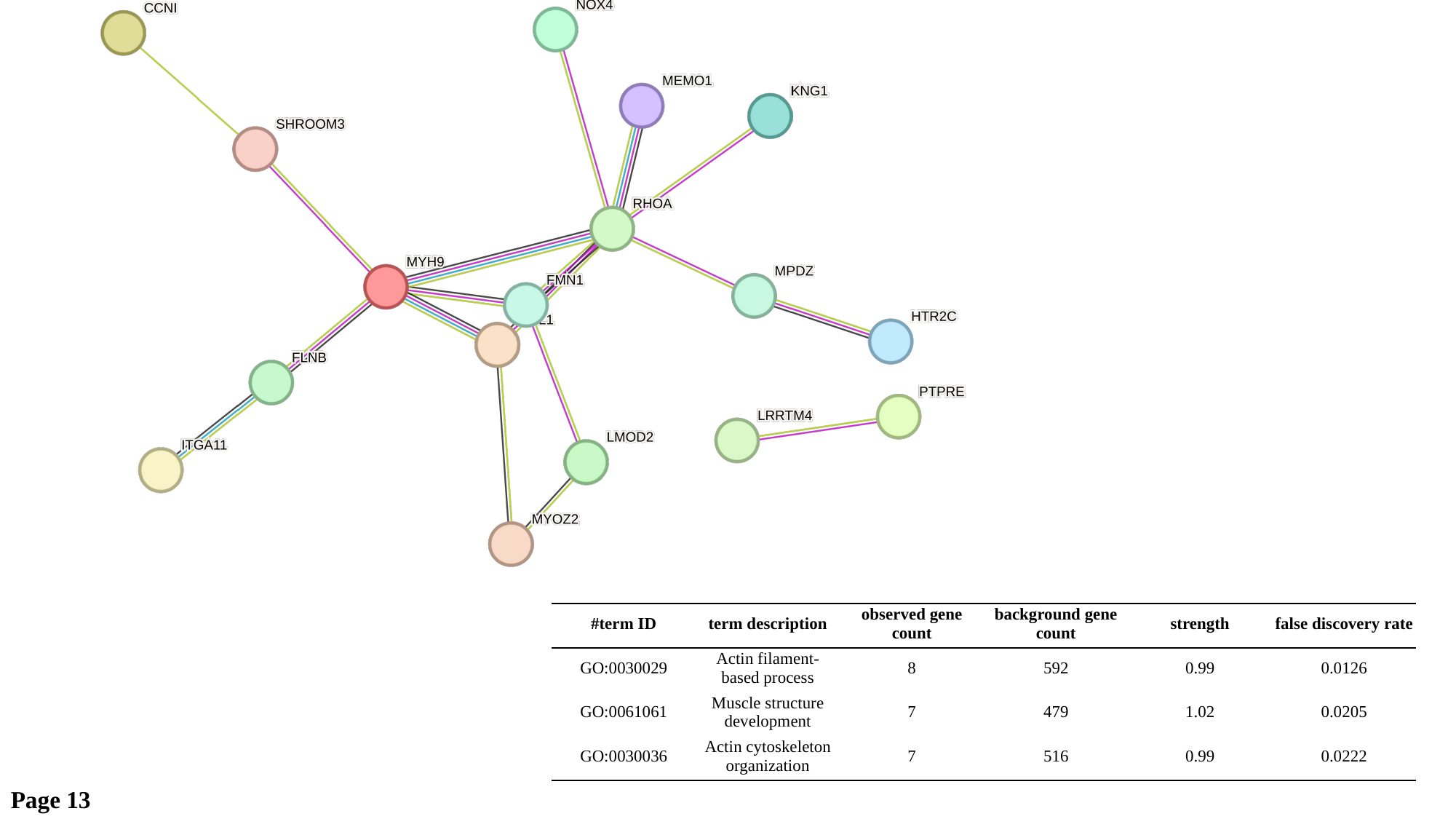

| #term ID | term description | observed gene count | background gene count | strength | false discovery rate |
| --- | --- | --- | --- | --- | --- |
| GO:0030029 | Actin filament-based process | 8 | 592 | 0.99 | 0.0126 |
| GO:0061061 | Muscle structure development | 7 | 479 | 1.02 | 0.0205 |
| GO:0030036 | Actin cytoskeleton organization | 7 | 516 | 0.99 | 0.0222 |
Page 13

## Slide 14
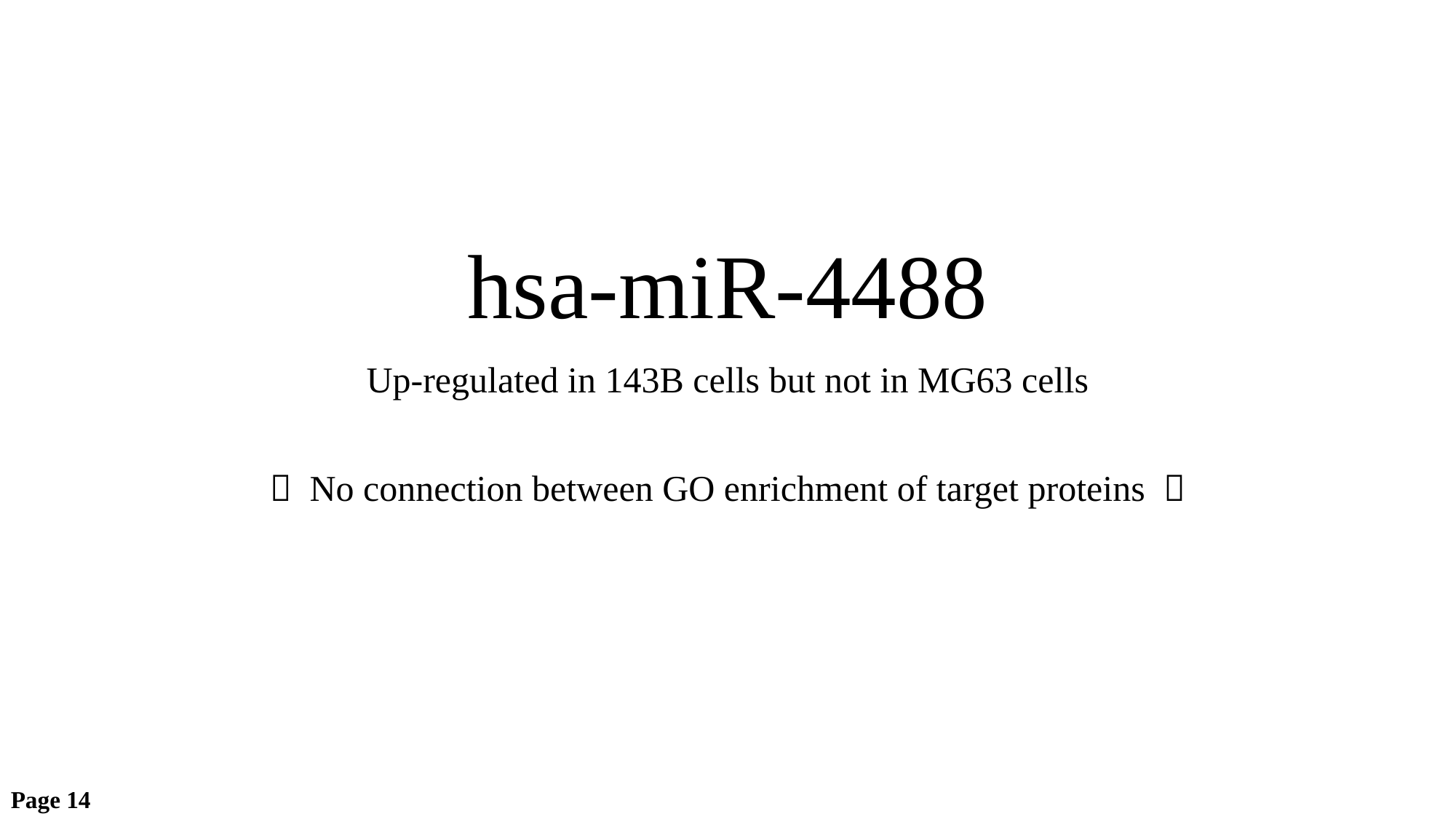

# hsa-miR-4488
Up-regulated in 143B cells but not in MG63 cells
（ No connection between GO enrichment of target proteins ）
Page 14

## Slide 15
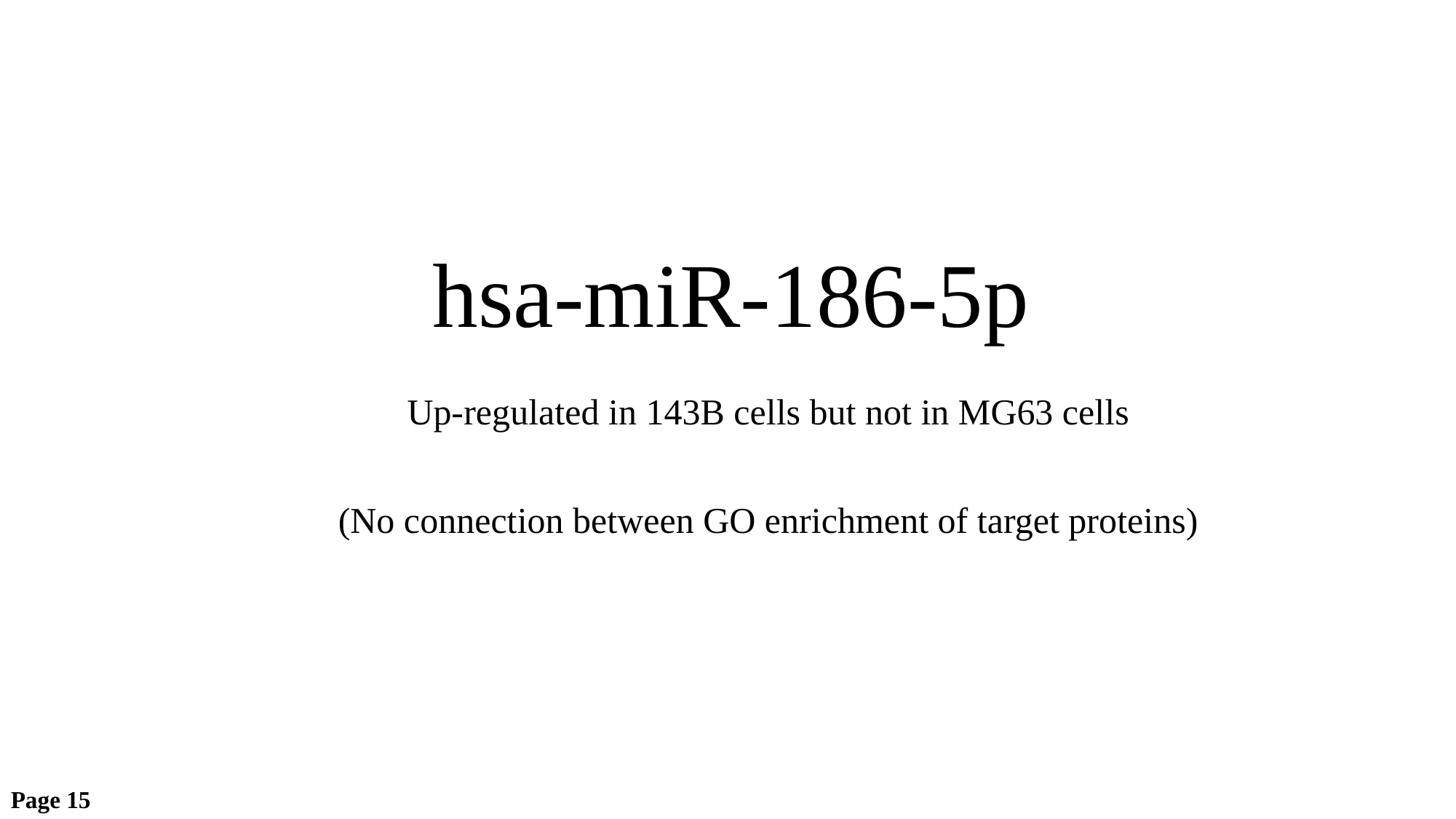

# hsa-miR-186-5p
Up-regulated in 143B cells but not in MG63 cells
(No connection between GO enrichment of target proteins)
Page 15

## Slide 16
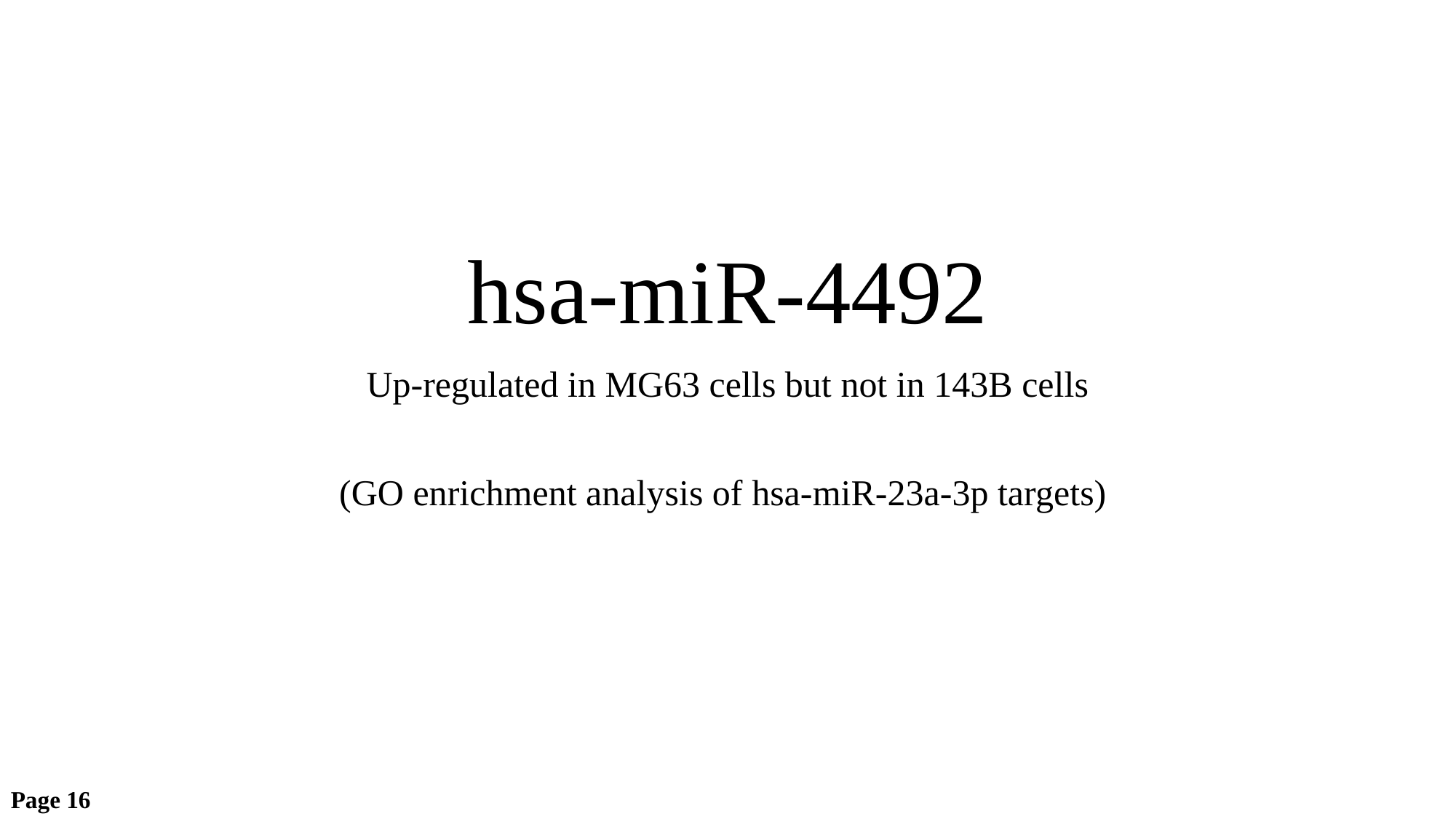

# hsa-miR-4492
Up-regulated in MG63 cells but not in 143B cells
(GO enrichment analysis of hsa-miR-23a-3p targets)
Page 16

## Slide 17
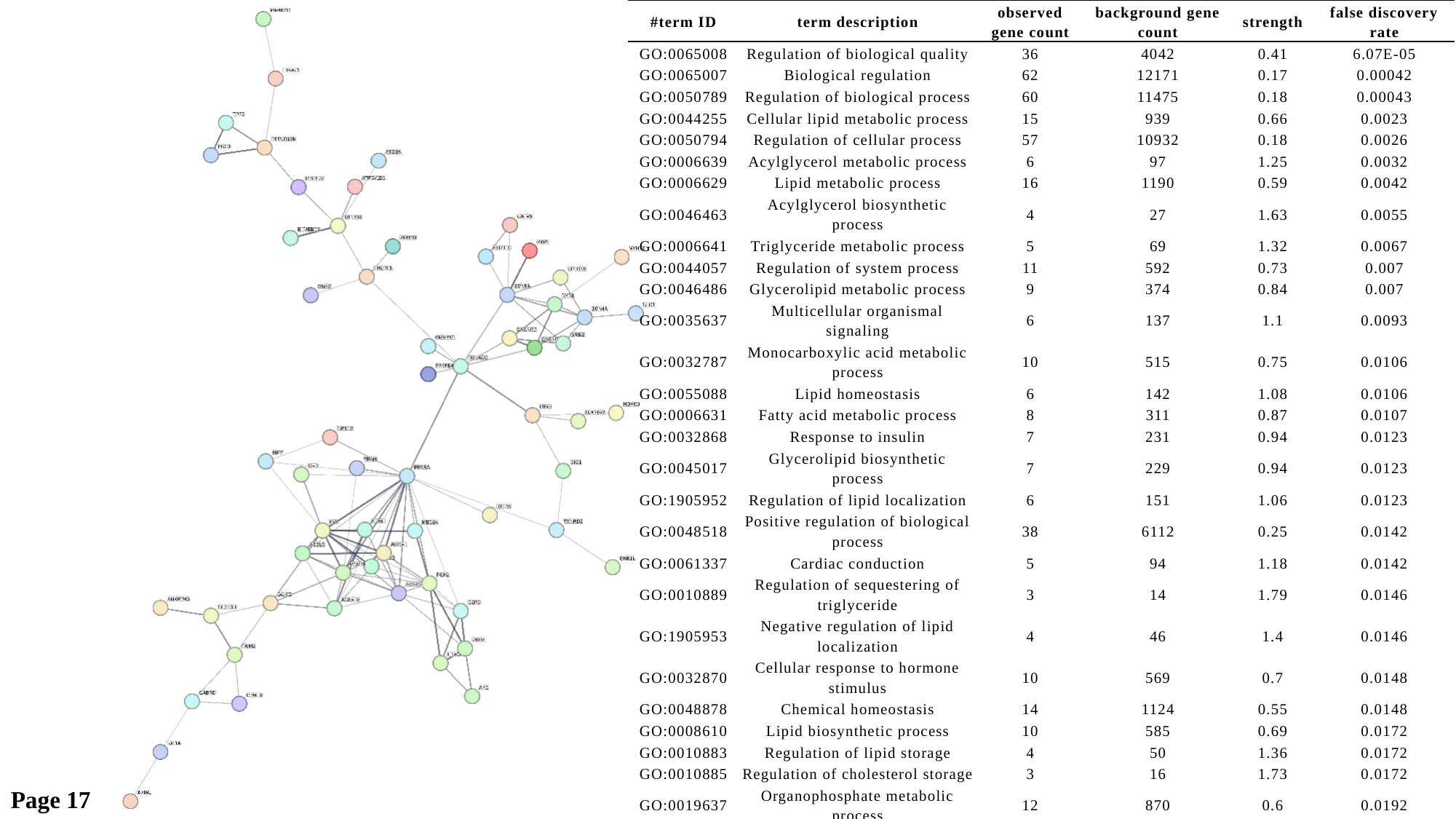

| #term ID | term description | observed gene count | background gene count | strength | false discovery rate |
| --- | --- | --- | --- | --- | --- |
| GO:0065008 | Regulation of biological quality | 36 | 4042 | 0.41 | 6.07E-05 |
| GO:0065007 | Biological regulation | 62 | 12171 | 0.17 | 0.00042 |
| GO:0050789 | Regulation of biological process | 60 | 11475 | 0.18 | 0.00043 |
| GO:0044255 | Cellular lipid metabolic process | 15 | 939 | 0.66 | 0.0023 |
| GO:0050794 | Regulation of cellular process | 57 | 10932 | 0.18 | 0.0026 |
| GO:0006639 | Acylglycerol metabolic process | 6 | 97 | 1.25 | 0.0032 |
| GO:0006629 | Lipid metabolic process | 16 | 1190 | 0.59 | 0.0042 |
| GO:0046463 | Acylglycerol biosynthetic process | 4 | 27 | 1.63 | 0.0055 |
| GO:0006641 | Triglyceride metabolic process | 5 | 69 | 1.32 | 0.0067 |
| GO:0044057 | Regulation of system process | 11 | 592 | 0.73 | 0.007 |
| GO:0046486 | Glycerolipid metabolic process | 9 | 374 | 0.84 | 0.007 |
| GO:0035637 | Multicellular organismal signaling | 6 | 137 | 1.1 | 0.0093 |
| GO:0032787 | Monocarboxylic acid metabolic process | 10 | 515 | 0.75 | 0.0106 |
| GO:0055088 | Lipid homeostasis | 6 | 142 | 1.08 | 0.0106 |
| GO:0006631 | Fatty acid metabolic process | 8 | 311 | 0.87 | 0.0107 |
| GO:0032868 | Response to insulin | 7 | 231 | 0.94 | 0.0123 |
| GO:0045017 | Glycerolipid biosynthetic process | 7 | 229 | 0.94 | 0.0123 |
| GO:1905952 | Regulation of lipid localization | 6 | 151 | 1.06 | 0.0123 |
| GO:0048518 | Positive regulation of biological process | 38 | 6112 | 0.25 | 0.0142 |
| GO:0061337 | Cardiac conduction | 5 | 94 | 1.18 | 0.0142 |
| GO:0010889 | Regulation of sequestering of triglyceride | 3 | 14 | 1.79 | 0.0146 |
| GO:1905953 | Negative regulation of lipid localization | 4 | 46 | 1.4 | 0.0146 |
| GO:0032870 | Cellular response to hormone stimulus | 10 | 569 | 0.7 | 0.0148 |
| GO:0048878 | Chemical homeostasis | 14 | 1124 | 0.55 | 0.0148 |
| GO:0008610 | Lipid biosynthetic process | 10 | 585 | 0.69 | 0.0172 |
| GO:0010883 | Regulation of lipid storage | 4 | 50 | 1.36 | 0.0172 |
| GO:0010885 | Regulation of cholesterol storage | 3 | 16 | 1.73 | 0.0172 |
| GO:0019637 | Organophosphate metabolic process | 12 | 870 | 0.6 | 0.0192 |
| GO:0042592 | Homeostatic process | 17 | 1676 | 0.47 | 0.0197 |
| GO:0009893 | Positive regulation of metabolic process | 28 | 3893 | 0.32 | 0.0198 |
| GO:0071495 | Cellular response to endogenous stimulus | 14 | 1181 | 0.53 | 0.0198 |
| GO:0019432 | Triglyceride biosynthetic process | 3 | 20 | 1.64 | 0.0253 |
| GO:0043434 | Response to peptide hormone | 8 | 394 | 0.77 | 0.026 |
| GO:0044242 | Cellular lipid catabolic process | 6 | 196 | 0.94 | 0.026 |
| GO:0010888 | Negative regulation of lipid storage | 3 | 21 | 1.61 | 0.0265 |
| GO:0051004 | Regulation of lipoprotein lipase activity | 3 | 22 | 1.59 | 0.0293 |
| GO:0019433 | Triglyceride catabolic process | 3 | 23 | 1.57 | 0.0321 |
| GO:0023052 | Signaling | 33 | 5239 | 0.26 | 0.0345 |
| GO:0090407 | Organophosphate biosynthetic process | 9 | 539 | 0.68 | 0.0345 |
| GO:0009719 | Response to endogenous stimulus | 15 | 1447 | 0.47 | 0.0352 |
| GO:0009058 | Biosynthetic process | 22 | 2788 | 0.36 | 0.0366 |
| GO:0055022 | Negative regulation of cardiac muscle tissue growth | 3 | 25 | 1.54 | 0.0366 |
| GO:0010614 | Negative regulation of cardiac muscle hypertrophy | 3 | 26 | 1.52 | 0.038 |
| GO:0007154 | Cell communication | 33 | 5320 | 0.25 | 0.0401 |
| GO:0010243 | Response to organonitrogen compound | 12 | 987 | 0.54 | 0.0401 |
| GO:0086019 | Cell-cell signaling involved in cardiac conduction | 3 | 27 | 1.5 | 0.0401 |
| GO:1900740 | Positive regulation of protein insertion into mitochondrial membrane involved in apoptotic signaling pathway | 3 | 27 | 1.5 | 0.0401 |
| GO:0009725 | Response to hormone | 11 | 849 | 0.57 | 0.041 |
| GO:0090257 | Regulation of muscle system process | 6 | 231 | 0.87 | 0.041 |
| GO:1901653 | Cellular response to peptide | 7 | 331 | 0.78 | 0.041 |
| GO:0034220 | Ion transmembrane transport | 12 | 1010 | 0.53 | 0.0416 |
| GO:0098660 | Inorganic ion transmembrane transport | 10 | 714 | 0.61 | 0.0416 |
| GO:0006811 | Ion transport | 14 | 1344 | 0.48 | 0.0427 |
| GO:0071417 | Cellular response to organonitrogen compound | 9 | 590 | 0.64 | 0.0465 |
| GO:0010743 | Regulation of macrophage derived foam cell differentiation | 3 | 31 | 1.44 | 0.0471 |
| GO:0043502 | Regulation of muscle adaptation | 4 | 82 | 1.15 | 0.0475 |
| GO:0008016 | Regulation of heart contraction | 6 | 245 | 0.85 | 0.0481 |
Page 17

## Slide 18
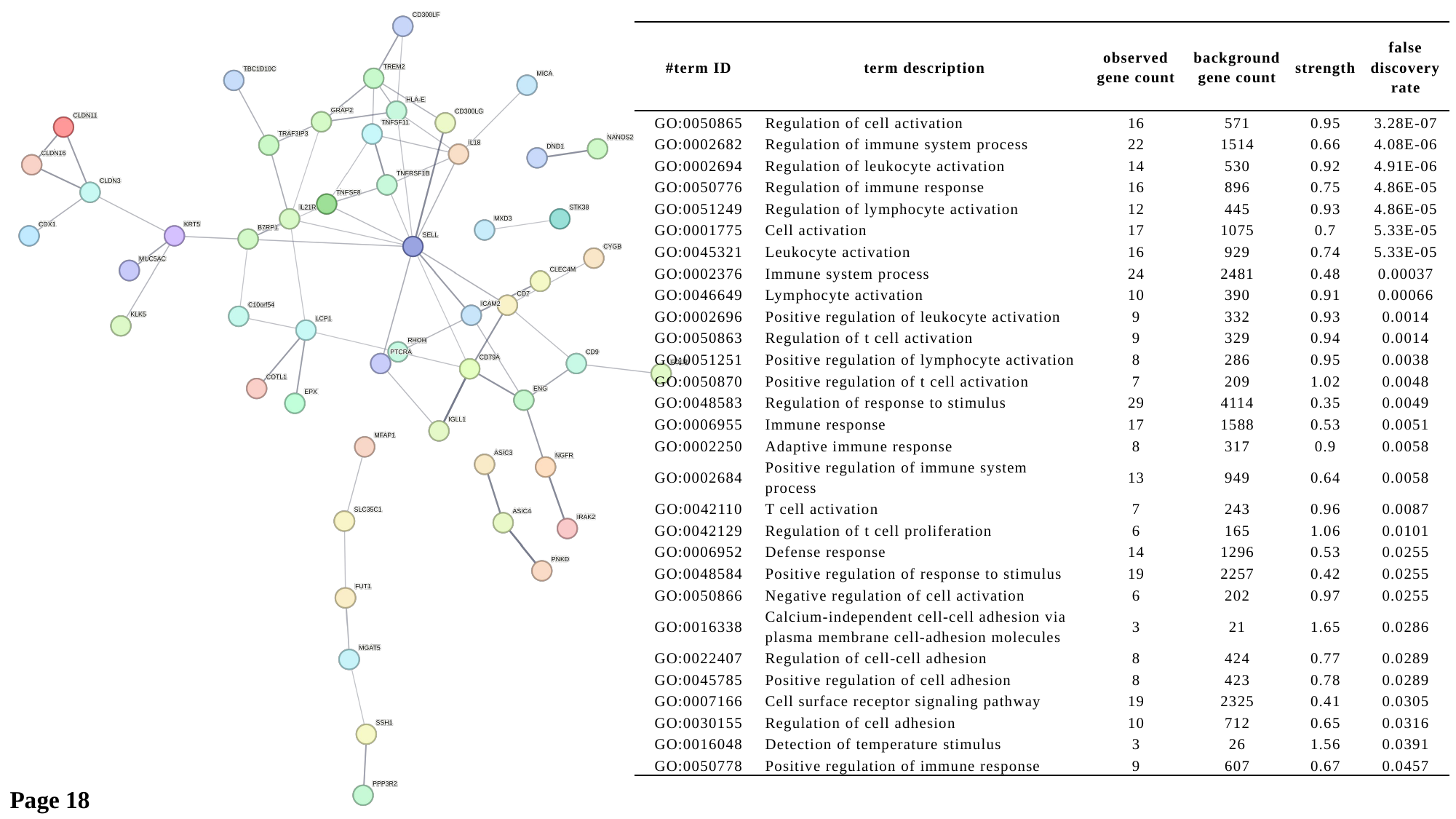

| #term ID | term description | observed gene count | background gene count | strength | false discovery rate |
| --- | --- | --- | --- | --- | --- |
| GO:0050865 | Regulation of cell activation | 16 | 571 | 0.95 | 3.28E-07 |
| GO:0002682 | Regulation of immune system process | 22 | 1514 | 0.66 | 4.08E-06 |
| GO:0002694 | Regulation of leukocyte activation | 14 | 530 | 0.92 | 4.91E-06 |
| GO:0050776 | Regulation of immune response | 16 | 896 | 0.75 | 4.86E-05 |
| GO:0051249 | Regulation of lymphocyte activation | 12 | 445 | 0.93 | 4.86E-05 |
| GO:0001775 | Cell activation | 17 | 1075 | 0.7 | 5.33E-05 |
| GO:0045321 | Leukocyte activation | 16 | 929 | 0.74 | 5.33E-05 |
| GO:0002376 | Immune system process | 24 | 2481 | 0.48 | 0.00037 |
| GO:0046649 | Lymphocyte activation | 10 | 390 | 0.91 | 0.00066 |
| GO:0002696 | Positive regulation of leukocyte activation | 9 | 332 | 0.93 | 0.0014 |
| GO:0050863 | Regulation of t cell activation | 9 | 329 | 0.94 | 0.0014 |
| GO:0051251 | Positive regulation of lymphocyte activation | 8 | 286 | 0.95 | 0.0038 |
| GO:0050870 | Positive regulation of t cell activation | 7 | 209 | 1.02 | 0.0048 |
| GO:0048583 | Regulation of response to stimulus | 29 | 4114 | 0.35 | 0.0049 |
| GO:0006955 | Immune response | 17 | 1588 | 0.53 | 0.0051 |
| GO:0002250 | Adaptive immune response | 8 | 317 | 0.9 | 0.0058 |
| GO:0002684 | Positive regulation of immune system process | 13 | 949 | 0.64 | 0.0058 |
| GO:0042110 | T cell activation | 7 | 243 | 0.96 | 0.0087 |
| GO:0042129 | Regulation of t cell proliferation | 6 | 165 | 1.06 | 0.0101 |
| GO:0006952 | Defense response | 14 | 1296 | 0.53 | 0.0255 |
| GO:0048584 | Positive regulation of response to stimulus | 19 | 2257 | 0.42 | 0.0255 |
| GO:0050866 | Negative regulation of cell activation | 6 | 202 | 0.97 | 0.0255 |
| GO:0016338 | Calcium-independent cell-cell adhesion via plasma membrane cell-adhesion molecules | 3 | 21 | 1.65 | 0.0286 |
| GO:0022407 | Regulation of cell-cell adhesion | 8 | 424 | 0.77 | 0.0289 |
| GO:0045785 | Positive regulation of cell adhesion | 8 | 423 | 0.78 | 0.0289 |
| GO:0007166 | Cell surface receptor signaling pathway | 19 | 2325 | 0.41 | 0.0305 |
| GO:0030155 | Regulation of cell adhesion | 10 | 712 | 0.65 | 0.0316 |
| GO:0016048 | Detection of temperature stimulus | 3 | 26 | 1.56 | 0.0391 |
| GO:0050778 | Positive regulation of immune response | 9 | 607 | 0.67 | 0.0457 |
Page 18

## Slide 19
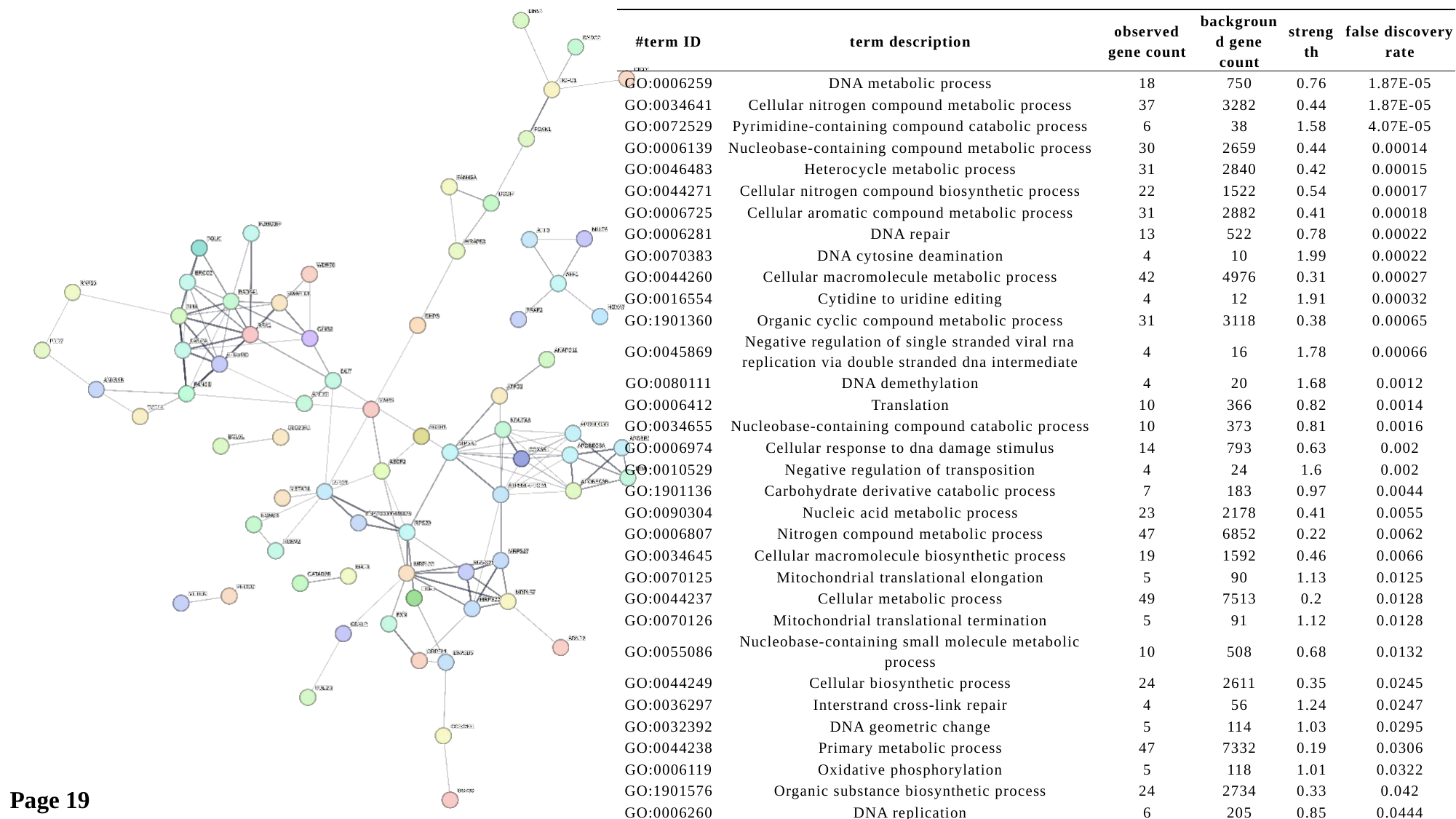

| #term ID | term description | observed gene count | background gene count | strength | false discovery rate |
| --- | --- | --- | --- | --- | --- |
| GO:0006259 | DNA metabolic process | 18 | 750 | 0.76 | 1.87E-05 |
| GO:0034641 | Cellular nitrogen compound metabolic process | 37 | 3282 | 0.44 | 1.87E-05 |
| GO:0072529 | Pyrimidine-containing compound catabolic process | 6 | 38 | 1.58 | 4.07E-05 |
| GO:0006139 | Nucleobase-containing compound metabolic process | 30 | 2659 | 0.44 | 0.00014 |
| GO:0046483 | Heterocycle metabolic process | 31 | 2840 | 0.42 | 0.00015 |
| GO:0044271 | Cellular nitrogen compound biosynthetic process | 22 | 1522 | 0.54 | 0.00017 |
| GO:0006725 | Cellular aromatic compound metabolic process | 31 | 2882 | 0.41 | 0.00018 |
| GO:0006281 | DNA repair | 13 | 522 | 0.78 | 0.00022 |
| GO:0070383 | DNA cytosine deamination | 4 | 10 | 1.99 | 0.00022 |
| GO:0044260 | Cellular macromolecule metabolic process | 42 | 4976 | 0.31 | 0.00027 |
| GO:0016554 | Cytidine to uridine editing | 4 | 12 | 1.91 | 0.00032 |
| GO:1901360 | Organic cyclic compound metabolic process | 31 | 3118 | 0.38 | 0.00065 |
| GO:0045869 | Negative regulation of single stranded viral rna replication via double stranded dna intermediate | 4 | 16 | 1.78 | 0.00066 |
| GO:0080111 | DNA demethylation | 4 | 20 | 1.68 | 0.0012 |
| GO:0006412 | Translation | 10 | 366 | 0.82 | 0.0014 |
| GO:0034655 | Nucleobase-containing compound catabolic process | 10 | 373 | 0.81 | 0.0016 |
| GO:0006974 | Cellular response to dna damage stimulus | 14 | 793 | 0.63 | 0.002 |
| GO:0010529 | Negative regulation of transposition | 4 | 24 | 1.6 | 0.002 |
| GO:1901136 | Carbohydrate derivative catabolic process | 7 | 183 | 0.97 | 0.0044 |
| GO:0090304 | Nucleic acid metabolic process | 23 | 2178 | 0.41 | 0.0055 |
| GO:0006807 | Nitrogen compound metabolic process | 47 | 6852 | 0.22 | 0.0062 |
| GO:0034645 | Cellular macromolecule biosynthetic process | 19 | 1592 | 0.46 | 0.0066 |
| GO:0070125 | Mitochondrial translational elongation | 5 | 90 | 1.13 | 0.0125 |
| GO:0044237 | Cellular metabolic process | 49 | 7513 | 0.2 | 0.0128 |
| GO:0070126 | Mitochondrial translational termination | 5 | 91 | 1.12 | 0.0128 |
| GO:0055086 | Nucleobase-containing small molecule metabolic process | 10 | 508 | 0.68 | 0.0132 |
| GO:0044249 | Cellular biosynthetic process | 24 | 2611 | 0.35 | 0.0245 |
| GO:0036297 | Interstrand cross-link repair | 4 | 56 | 1.24 | 0.0247 |
| GO:0032392 | DNA geometric change | 5 | 114 | 1.03 | 0.0295 |
| GO:0044238 | Primary metabolic process | 47 | 7332 | 0.19 | 0.0306 |
| GO:0006119 | Oxidative phosphorylation | 5 | 118 | 1.01 | 0.0322 |
| GO:1901576 | Organic substance biosynthetic process | 24 | 2734 | 0.33 | 0.042 |
| GO:0006260 | DNA replication | 6 | 205 | 0.85 | 0.0444 |
| GO:0046034 | ATP metabolic process | 6 | 204 | 0.85 | 0.0444 |
Page 19

## Slide 20
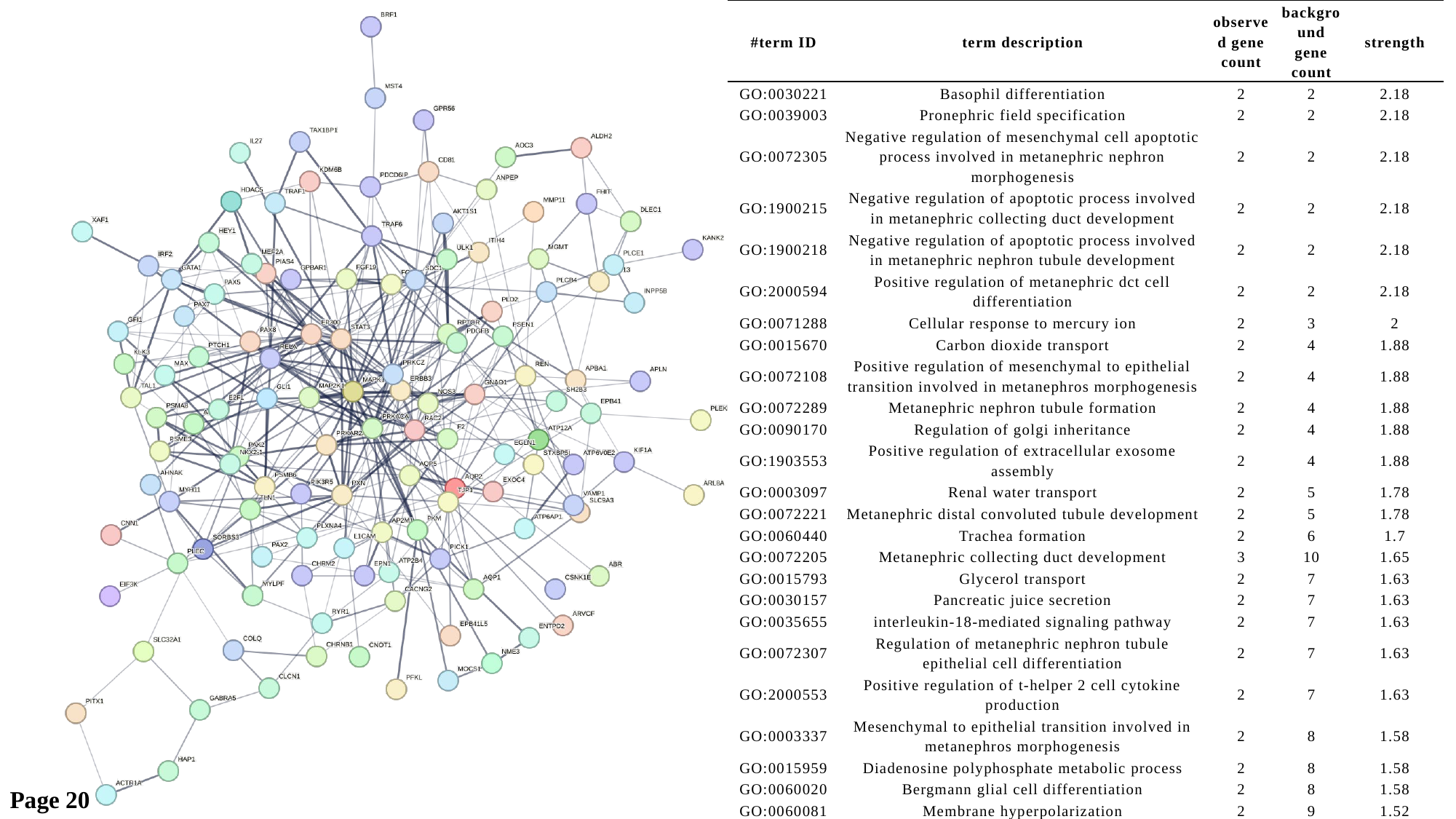

| #term ID | term description | observed gene count | background gene count | strength |
| --- | --- | --- | --- | --- |
| GO:0030221 | Basophil differentiation | 2 | 2 | 2.18 |
| GO:0039003 | Pronephric field specification | 2 | 2 | 2.18 |
| GO:0072305 | Negative regulation of mesenchymal cell apoptotic process involved in metanephric nephron morphogenesis | 2 | 2 | 2.18 |
| GO:1900215 | Negative regulation of apoptotic process involved in metanephric collecting duct development | 2 | 2 | 2.18 |
| GO:1900218 | Negative regulation of apoptotic process involved in metanephric nephron tubule development | 2 | 2 | 2.18 |
| GO:2000594 | Positive regulation of metanephric dct cell differentiation | 2 | 2 | 2.18 |
| GO:0071288 | Cellular response to mercury ion | 2 | 3 | 2 |
| GO:0015670 | Carbon dioxide transport | 2 | 4 | 1.88 |
| GO:0072108 | Positive regulation of mesenchymal to epithelial transition involved in metanephros morphogenesis | 2 | 4 | 1.88 |
| GO:0072289 | Metanephric nephron tubule formation | 2 | 4 | 1.88 |
| GO:0090170 | Regulation of golgi inheritance | 2 | 4 | 1.88 |
| GO:1903553 | Positive regulation of extracellular exosome assembly | 2 | 4 | 1.88 |
| GO:0003097 | Renal water transport | 2 | 5 | 1.78 |
| GO:0072221 | Metanephric distal convoluted tubule development | 2 | 5 | 1.78 |
| GO:0060440 | Trachea formation | 2 | 6 | 1.7 |
| GO:0072205 | Metanephric collecting duct development | 3 | 10 | 1.65 |
| GO:0015793 | Glycerol transport | 2 | 7 | 1.63 |
| GO:0030157 | Pancreatic juice secretion | 2 | 7 | 1.63 |
| GO:0035655 | interleukin-18-mediated signaling pathway | 2 | 7 | 1.63 |
| GO:0072307 | Regulation of metanephric nephron tubule epithelial cell differentiation | 2 | 7 | 1.63 |
| GO:2000553 | Positive regulation of t-helper 2 cell cytokine production | 2 | 7 | 1.63 |
| GO:0003337 | Mesenchymal to epithelial transition involved in metanephros morphogenesis | 2 | 8 | 1.58 |
| GO:0015959 | Diadenosine polyphosphate metabolic process | 2 | 8 | 1.58 |
| GO:0060020 | Bergmann glial cell differentiation | 2 | 8 | 1.58 |
| GO:0060081 | Membrane hyperpolarization | 2 | 9 | 1.52 |
| GO:0099527 | Postsynapse to nucleus signaling pathway | 2 | 9 | 1.52 |
| GO:0072216 | Positive regulation of metanephros development | 3 | 14 | 1.51 |
| GO:0010544 | Negative regulation of platelet activation | 4 | 19 | 1.5 |
| GO:0070528 | Protein kinase c signaling | 3 | 15 | 1.48 |
| GO:0030219 | Megakaryocyte differentiation | 4 | 21 | 1.46 |
| GO:0030878 | Thyroid gland development | 4 | 21 | 1.46 |
| GO:0021904 | Dorsal/ventral neural tube patterning | 3 | 17 | 1.42 |
| GO:0035855 | Megakaryocyte development | 3 | 17 | 1.42 |
| GO:0031293 | Membrane protein intracellular domain proteolysis | 3 | 18 | 1.4 |
| GO:0072202 | Cell differentiation involved in metanephros development | 3 | 18 | 1.4 |
| GO:0030220 | Platelet formation | 3 | 19 | 1.38 |
| GO:0002726 | Positive regulation of t cell cytokine production | 3 | 20 | 1.35 |
| GO:1904996 | Positive regulation of leukocyte adhesion to vascular endothelial cell | 3 | 20 | 1.35 |
| GO:0035162 | Embryonic hemopoiesis | 3 | 22 | 1.31 |
| GO:2000678 | Negative regulation of transcription regulatory region dna binding | 3 | 23 | 1.29 |
| GO:2000810 | Regulation of bicellular tight junction assembly | 3 | 23 | 1.29 |
| GO:2000036 | Regulation of stem cell population maintenance | 4 | 33 | 1.26 |
| GO:0048333 | Mesodermal cell differentiation | 3 | 26 | 1.24 |
| GO:0048645 | Animal organ formation | 4 | 36 | 1.22 |
| GO:0002026 | Regulation of the force of heart contraction | 3 | 27 | 1.22 |
| GO:1903671 | Negative regulation of sprouting angiogenesis | 3 | 27 | 1.22 |
| GO:0003091 | Renal water homeostasis | 4 | 37 | 1.21 |
| GO:0070168 | Negative regulation of biomineral tissue development | 3 | 28 | 1.21 |
| GO:1902895 | Positive regulation of pri-mirna transcription by rna polymerase ii | 4 | 40 | 1.18 |
| GO:0072210 | Metanephric nephron development | 3 | 31 | 1.16 |
| GO:0097028 | Dendritic cell differentiation | 3 | 31 | 1.16 |
| GO:0045648 | Positive regulation of erythrocyte differentiation | 3 | 32 | 1.15 |
| GO:0048708 | Astrocyte differentiation | 5 | 55 | 1.14 |
| GO:0061005 | Cell differentiation involved in kidney development | 4 | 44 | 1.14 |
| GO:0035850 | Epithelial cell differentiation involved in kidney development | 3 | 33 | 1.14 |
| GO:0060674 | Placenta blood vessel development | 3 | 33 | 1.14 |
| GO:0001707 | Mesoderm formation | 6 | 67 | 1.13 |
| GO:2000677 | Regulation of transcription regulatory region dna binding | 5 | 56 | 1.13 |
| GO:0043403 | Skeletal muscle tissue regeneration | 3 | 34 | 1.12 |
| GO:0048821 | Erythrocyte development | 3 | 34 | 1.12 |
| GO:0051354 | Negative regulation of oxidoreductase activity | 3 | 34 | 1.12 |
| GO:0007271 | Synaptic transmission, cholinergic | 3 | 35 | 1.11 |
| GO:0098926 | Postsynaptic signal transduction | 3 | 36 | 1.1 |
| GO:0060711 | Labyrinthine layer development | 4 | 49 | 1.09 |
| GO:0030865 | Cortical cytoskeleton organization | 4 | 53 | 1.06 |
| GO:0002223 | Stimulatory c-type lectin receptor signaling pathway | 8 | 111 | 1.04 |
| GO:0061045 | Negative regulation of wound healing | 5 | 70 | 1.03 |
| GO:1902117 | Positive regulation of organelle assembly | 5 | 71 | 1.03 |
| GO:0043392 | Negative regulation of dna binding | 4 | 56 | 1.03 |
| GO:1903670 | Regulation of sprouting angiogenesis | 5 | 72 | 1.02 |
| GO:1902036 | Regulation of hematopoietic stem cell differentiation | 5 | 74 | 1.01 |
| GO:0051289 | Protein homotetramerization | 4 | 60 | 1 |
| GO:0061515 | Myeloid cell development | 4 | 60 | 1 |
| GO:0031032 | Actomyosin structure organization | 7 | 111 | 0.98 |
| GO:0001938 | Positive regulation of endothelial cell proliferation | 6 | 94 | 0.98 |
| GO:0061418 | Regulation of transcription from rna polymerase ii promoter in response to hypoxia | 5 | 78 | 0.98 |
| GO:0038061 | NIK/NF-kappaB signaling | 5 | 81 | 0.97 |
| GO:0006521 | Regulation of cellular amino acid metabolic process | 4 | 64 | 0.97 |
| GO:0038095 | Fc-epsilon receptor signaling pathway | 7 | 115 | 0.96 |
| GO:0009953 | Dorsal/ventral pattern formation | 5 | 83 | 0.96 |
| GO:0048017 | Inositol lipid-mediated signaling | 5 | 83 | 0.96 |
| GO:0008088 | Axo-dendritic transport | 4 | 66 | 0.96 |
Page 20

## Slide 21
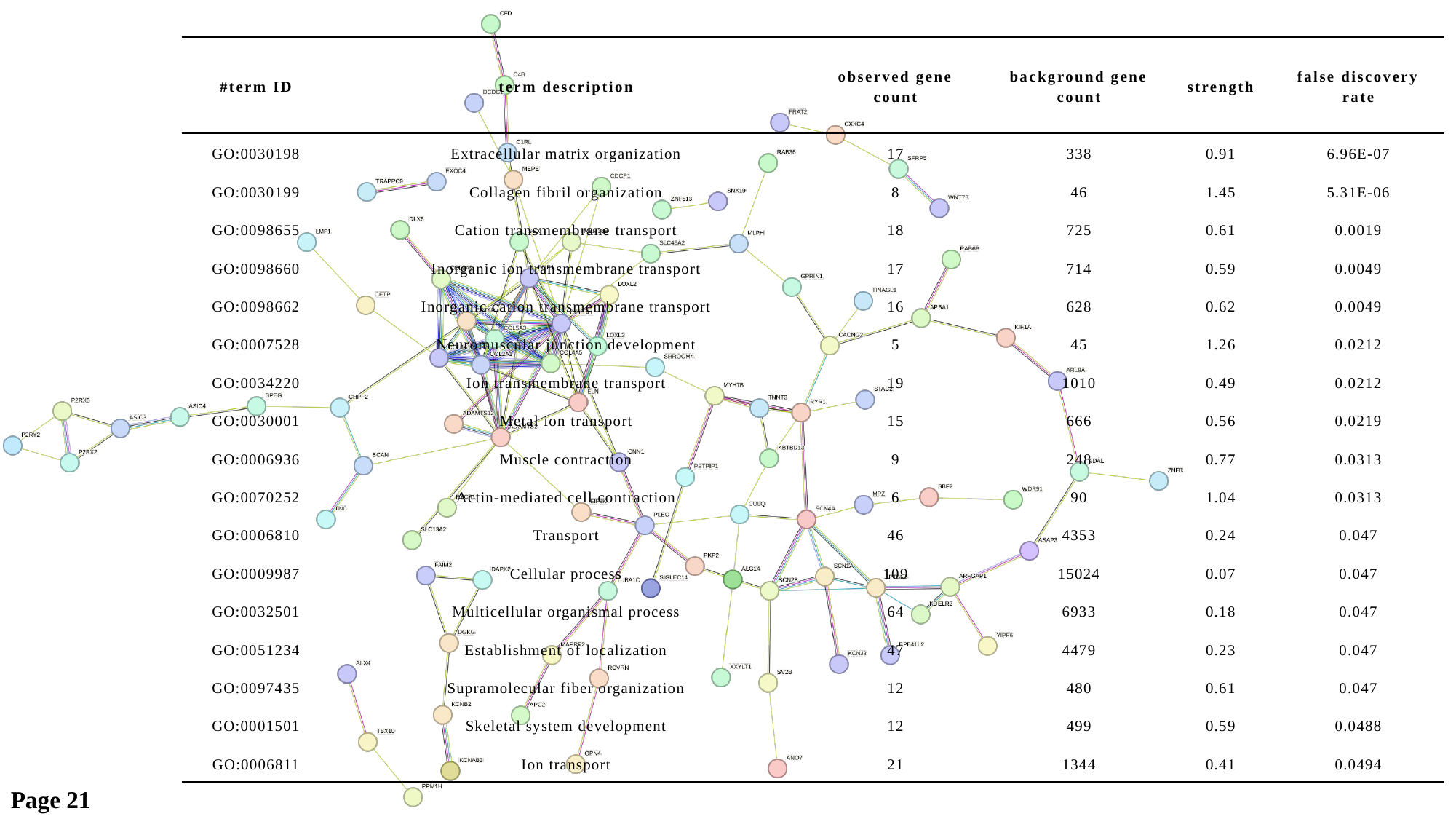

| #term ID | term description | observed gene count | background gene count | strength | false discovery rate |
| --- | --- | --- | --- | --- | --- |
| GO:0030198 | Extracellular matrix organization | 17 | 338 | 0.91 | 6.96E-07 |
| GO:0030199 | Collagen fibril organization | 8 | 46 | 1.45 | 5.31E-06 |
| GO:0098655 | Cation transmembrane transport | 18 | 725 | 0.61 | 0.0019 |
| GO:0098660 | Inorganic ion transmembrane transport | 17 | 714 | 0.59 | 0.0049 |
| GO:0098662 | Inorganic cation transmembrane transport | 16 | 628 | 0.62 | 0.0049 |
| GO:0007528 | Neuromuscular junction development | 5 | 45 | 1.26 | 0.0212 |
| GO:0034220 | Ion transmembrane transport | 19 | 1010 | 0.49 | 0.0212 |
| GO:0030001 | Metal ion transport | 15 | 666 | 0.56 | 0.0219 |
| GO:0006936 | Muscle contraction | 9 | 248 | 0.77 | 0.0313 |
| GO:0070252 | Actin-mediated cell contraction | 6 | 90 | 1.04 | 0.0313 |
| GO:0006810 | Transport | 46 | 4353 | 0.24 | 0.047 |
| GO:0009987 | Cellular process | 109 | 15024 | 0.07 | 0.047 |
| GO:0032501 | Multicellular organismal process | 64 | 6933 | 0.18 | 0.047 |
| GO:0051234 | Establishment of localization | 47 | 4479 | 0.23 | 0.047 |
| GO:0097435 | Supramolecular fiber organization | 12 | 480 | 0.61 | 0.047 |
| GO:0001501 | Skeletal system development | 12 | 499 | 0.59 | 0.0488 |
| GO:0006811 | Ion transport | 21 | 1344 | 0.41 | 0.0494 |
Page 21

## Slide 22
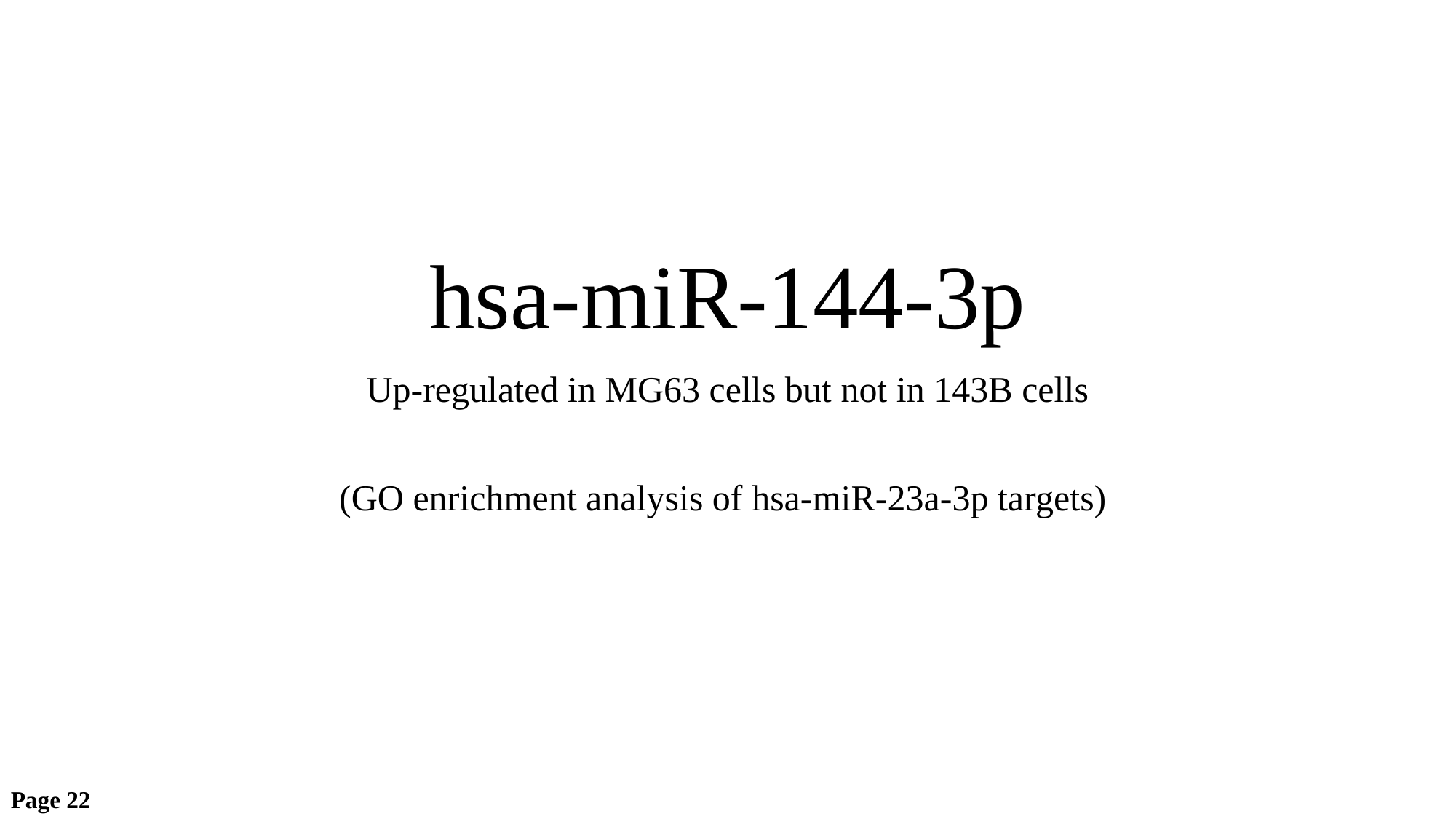

# hsa-miR-144-3p
Up-regulated in MG63 cells but not in 143B cells
(GO enrichment analysis of hsa-miR-23a-3p targets)
Page 22

## Slide 23
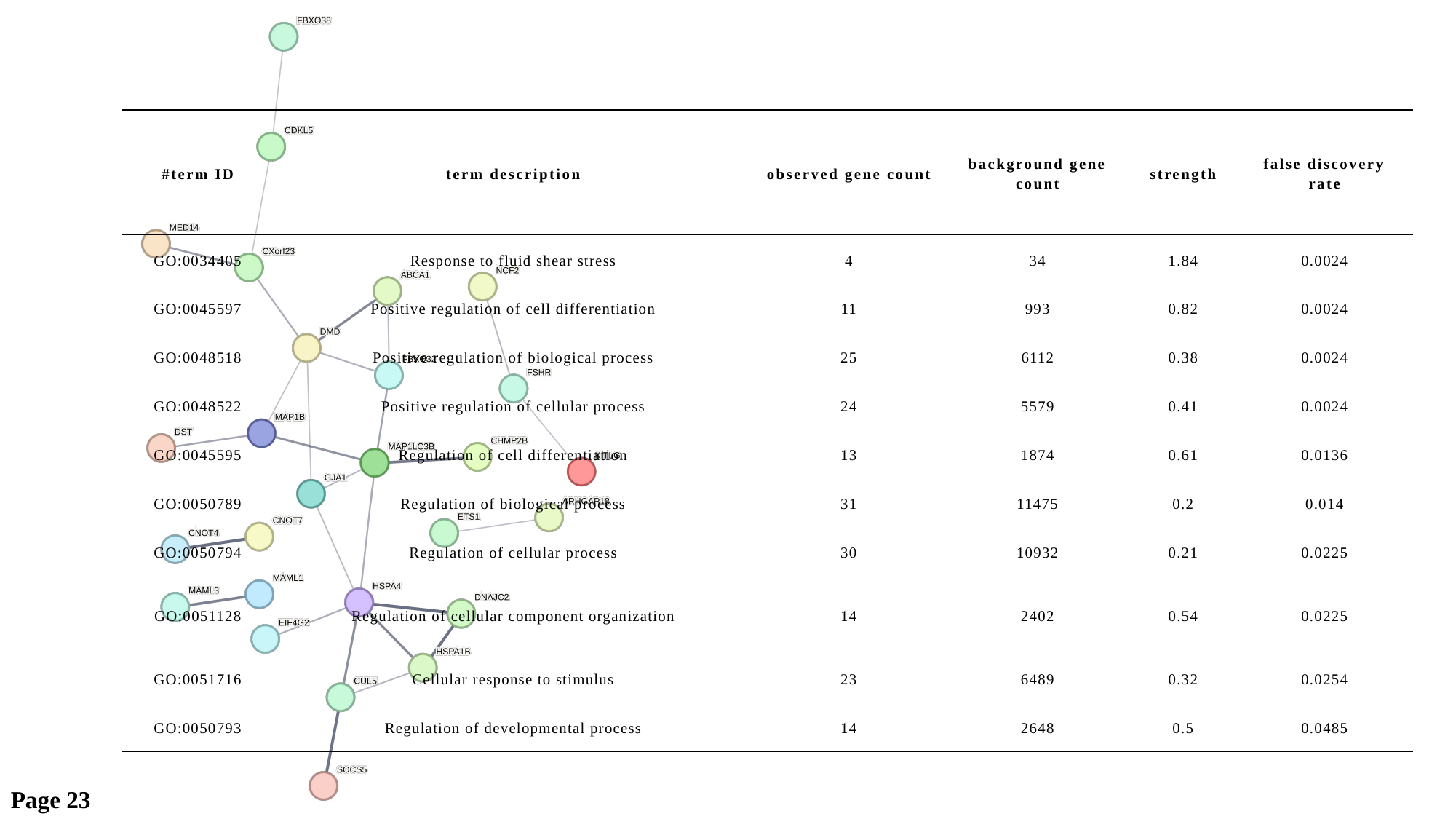

| #term ID | term description | observed gene count | background gene count | strength | false discovery rate |
| --- | --- | --- | --- | --- | --- |
| GO:0034405 | Response to fluid shear stress | 4 | 34 | 1.84 | 0.0024 |
| GO:0045597 | Positive regulation of cell differentiation | 11 | 993 | 0.82 | 0.0024 |
| GO:0048518 | Positive regulation of biological process | 25 | 6112 | 0.38 | 0.0024 |
| GO:0048522 | Positive regulation of cellular process | 24 | 5579 | 0.41 | 0.0024 |
| GO:0045595 | Regulation of cell differentiation | 13 | 1874 | 0.61 | 0.0136 |
| GO:0050789 | Regulation of biological process | 31 | 11475 | 0.2 | 0.014 |
| GO:0050794 | Regulation of cellular process | 30 | 10932 | 0.21 | 0.0225 |
| GO:0051128 | Regulation of cellular component organization | 14 | 2402 | 0.54 | 0.0225 |
| GO:0051716 | Cellular response to stimulus | 23 | 6489 | 0.32 | 0.0254 |
| GO:0050793 | Regulation of developmental process | 14 | 2648 | 0.5 | 0.0485 |
Page 23

## Slide 24
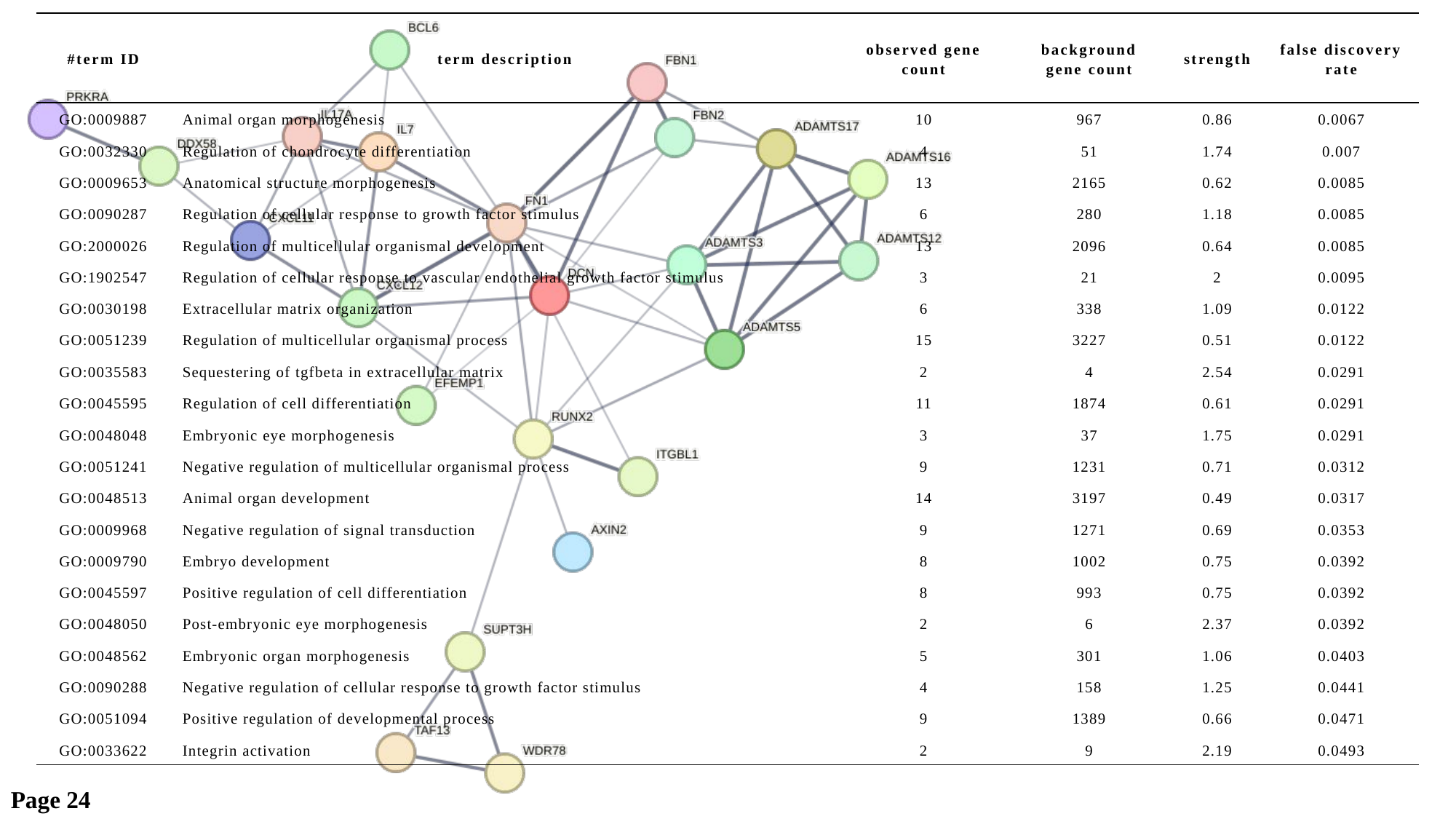

| #term ID | term description | observed gene count | background gene count | strength | false discovery rate |
| --- | --- | --- | --- | --- | --- |
| GO:0009887 | Animal organ morphogenesis | 10 | 967 | 0.86 | 0.0067 |
| GO:0032330 | Regulation of chondrocyte differentiation | 4 | 51 | 1.74 | 0.007 |
| GO:0009653 | Anatomical structure morphogenesis | 13 | 2165 | 0.62 | 0.0085 |
| GO:0090287 | Regulation of cellular response to growth factor stimulus | 6 | 280 | 1.18 | 0.0085 |
| GO:2000026 | Regulation of multicellular organismal development | 13 | 2096 | 0.64 | 0.0085 |
| GO:1902547 | Regulation of cellular response to vascular endothelial growth factor stimulus | 3 | 21 | 2 | 0.0095 |
| GO:0030198 | Extracellular matrix organization | 6 | 338 | 1.09 | 0.0122 |
| GO:0051239 | Regulation of multicellular organismal process | 15 | 3227 | 0.51 | 0.0122 |
| GO:0035583 | Sequestering of tgfbeta in extracellular matrix | 2 | 4 | 2.54 | 0.0291 |
| GO:0045595 | Regulation of cell differentiation | 11 | 1874 | 0.61 | 0.0291 |
| GO:0048048 | Embryonic eye morphogenesis | 3 | 37 | 1.75 | 0.0291 |
| GO:0051241 | Negative regulation of multicellular organismal process | 9 | 1231 | 0.71 | 0.0312 |
| GO:0048513 | Animal organ development | 14 | 3197 | 0.49 | 0.0317 |
| GO:0009968 | Negative regulation of signal transduction | 9 | 1271 | 0.69 | 0.0353 |
| GO:0009790 | Embryo development | 8 | 1002 | 0.75 | 0.0392 |
| GO:0045597 | Positive regulation of cell differentiation | 8 | 993 | 0.75 | 0.0392 |
| GO:0048050 | Post-embryonic eye morphogenesis | 2 | 6 | 2.37 | 0.0392 |
| GO:0048562 | Embryonic organ morphogenesis | 5 | 301 | 1.06 | 0.0403 |
| GO:0090288 | Negative regulation of cellular response to growth factor stimulus | 4 | 158 | 1.25 | 0.0441 |
| GO:0051094 | Positive regulation of developmental process | 9 | 1389 | 0.66 | 0.0471 |
| GO:0033622 | Integrin activation | 2 | 9 | 2.19 | 0.0493 |
Page 24

## Slide 25
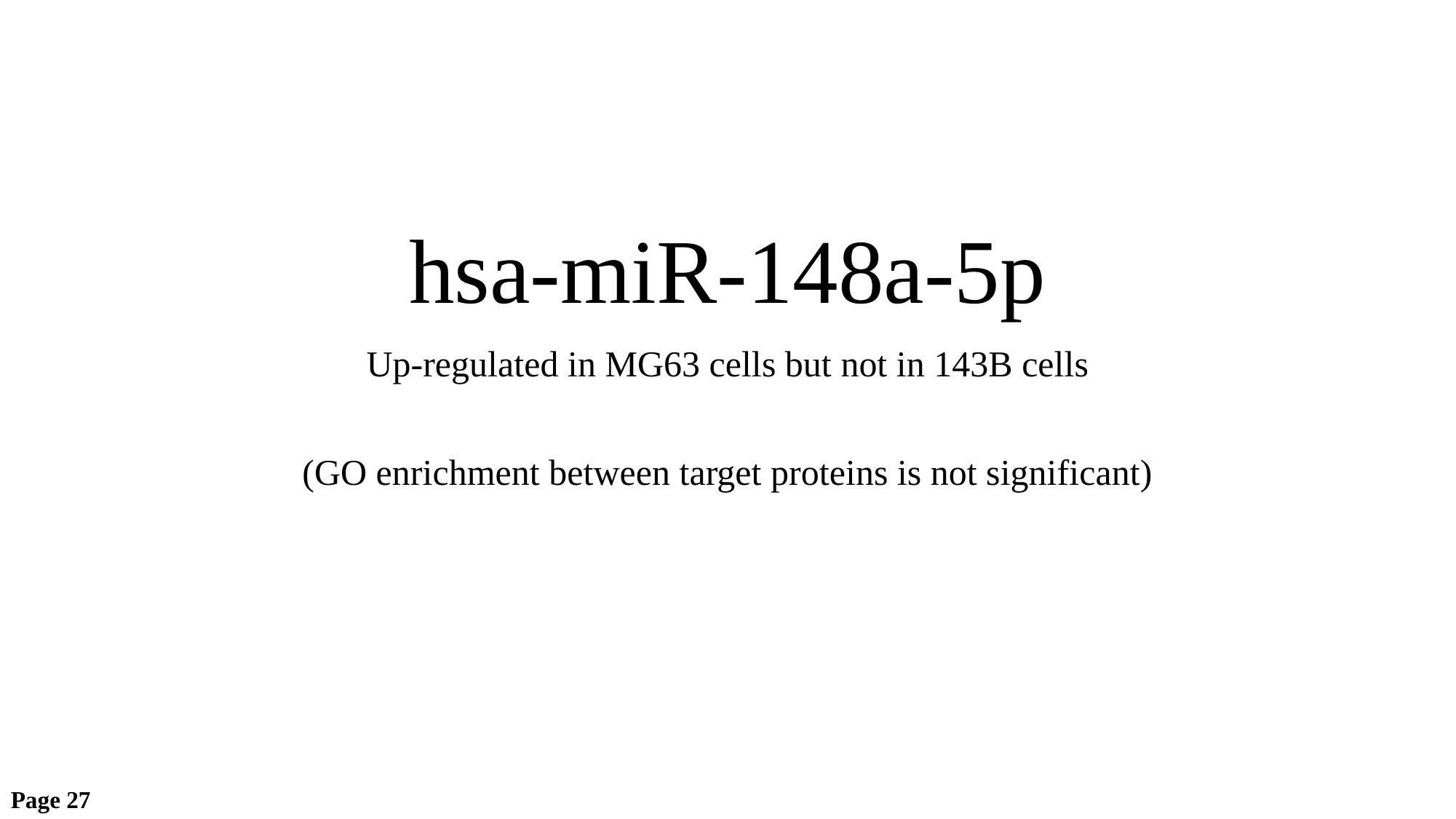

# hsa-miR-148a-5p
Up-regulated in MG63 cells but not in 143B cells
(GO enrichment between target proteins is not significant)
Page 27
